# Extracellular vesicles in infectious diseases caused by protozoan parasites in buffaloes

**DOI:** 10.1590/1678-9199-JVATITD-2019-0067

**Published:** 2020-05-29

**Authors:** Leticia Gomes de Pontes, Wanessa Fernanda Altei, Asier Galan, Petra Bilić, Nicolas Guillemin, Josipa Kuleš, Anita Horvatić, Lígia Nunes de Morais Ribeiro, Eneida de Paula, Virgínia Bodelão Richini Pereira, Simone Baldini Lucheis, Vladimir Mrljak, Peter David Eckersall, Rui Seabra Ferreira, Lucilene Delazari dos Santos

**Affiliations:** 1 Graduate Program in Tropical Diseases, Botucatu Medical School (FMB), São Paulo State University (UNESP), Botucatu, SP, Brazil.; 2 Laboratory of Biochemistry and Molecular Biology, Department of Physiological Sciences, Federal University of São Carlos (UFSCar), São Carlos, SP, Brazil.; 3 ERA Chair Team (VetMedZg), Clinic for Internal Diseases, Faculty of Veterinary Medicine, University of Zagreb, Zagreb, Croatia.; 4 Department of Biochemistry and Tissue Biology, Institute of Biology, University of Campinas (UNICAMP), Campinas, SP, Brazil.; 5 Adolfo Lutz Institute, Center of Regional Laboratories II, Bauru, SP, Brazil.; 6 Paulista Agency of Agribusiness Technology (APTA), Bauru, SP, Brazil.; 7 Institute of Biodiversity, Animal Health and Comparative Medicine, University of Glasgow, Glasgow, United Kingdom, UK.; 8 Graduate Program in Clinical Research, Botucatu Medical School (FMB), São Paulo State University (UNESP), Botucatu, SP, Brazil.; 9 Center for the Study of Venoms and Venomous Animals (CEVAP), São Paulo State University (UNESP), Botucatu, SP, Brazil.

**Keywords:** Extracellular vesicles, *Theileria* spp., Protozoan parasites, Nanoparticle tracking analysis, Proteomic analysis

## Abstract

**Background:**

Extracellular vesicles (EVs) are small membrane-bound vesicles of growing interest in vetetinary parasitology. The aim of the present report was to provide the first isolation, quantification and protein characterization of EVs from buffalo (*Bubalus bubalis)* sera infected with *Theileria* spp.

**Methods:**

Infected animals were identified through optical microscopy and PCR. EVs were isolated from buffalo sera by size-exclusion chromatography and characterized using western blotting analysis, nanoparticle tracking analysis and transmission electron microscopy. Subsequently, the proteins from isolated vesicles were characterized by mass spectrometry.

**Results:**

EVs from buffalo sera have shown sizes in the 124-140 nm range and 306 proteins were characterized. The protein-protein interaction analysis has evidenced biological processes and molecular function associated with signal transduction, binding, regulation of metabolic processes, transport, catalytic activity and response to acute stress. Five proteins have been shown to be differentially expressed between the control group and that infected with *Theileria* spp., all acting in the oxidative stress pathway.

**Conclusions:**

EVs from buffaloes infected with *Theileria* spp. were successfully isolated and characterized. This is an advance in the knowledge of host-parasite relationship that contributes to the understanding of host immune response and theileriosis evasion mechanisms. These findings may pave the way for searching new EVs candidate-markers for a better production of safe biological products derived from buffaloes.

## Background

Extracellular vesicles (EVs) are small vesicles known to play major roles in intercellular communication [1]. They are classified as small (SEVs) and large (LEVs) EVs based on their size, biogenesis and composition [2]. Exosomes, considered as SEVs, are 40-150-nm diameter vesicles originated in endosomal pathway. They emerge from budding of endosomal membranes, forming multivesicular endosomes (MVEs), further maturated to late endosomes or multivesicular bodies (MVBs) containing the intraluminal vesicles [3].

Exosomes have a lipid bilayer membrane enriched with host specific proteins, besides mRNA and microRNA that confers to them the capacity of transporting information from origin cell to a specific target [4]. These SEVs are considered as promising potential biomarkers in diagnosis, since their molecular composition reflects a “signature” of the origin cell [5,6]. In this way, after studies in tumor development [7], SEVs became attractive targets in several diseases, given their increased secretion in this context [8]. 

Parasitic diseases use vesicles to communicate with the host cells [9], where infected cells have the ability to change the composition and function of these released vesicles during host-parasite interactions, thus mediating the disease development [10]. Apicomplexa comprises a diverse group of obligate intracellular parasites, most known species are pathogens of humans and domestic animals. The hemoparasites *Theileria* spp. cause tick-borne diseases of great health and economic impacts, especially in tropical areas [11-15]. 

The potential clinical uses of EVs in human Apicomplexa infections have been extensively described [16,17]. However, there is limited data about the use of EVs for diseases affecting domestic and farm animals. The role of EVs in apicomplexan parasite infections is a prospective field, with great potential for biotechnological development targeting clinical and economic interests for veterinary medicine [18,19]. 

There is a need for innovative approaches that focus on theileriosis since there is no efficient treatment against it, despite its economic impact. EVs have been explored as a promising target to study parasite-host interactions in a level never accessed before. They represent a valuable source for new vaccines and diagnostic tools for neglected tropical and vector-borne diseases conjointly for animal health and production [20-22]. 

Buffaloes from *Bubalus bubalis* species have gained relevance in the world economy due to the quality of their milk, meat, and leather, in addition of being important donors of blood components. A heterologous fibrin sealant was developed from the fibrinogen-rich cryoprecipitate of buffalo blood [23,24], which has been used in several clinical applications such as in the treatment of chronic venous ulcers [25]. The use of fibrin has incresed due to its biological properties in skin tissue regeneration as well as wound healing processes [26]. 

A number of strategies employing synthetic polymers such as polyethylene glycol [27] and engineered hemostatic polymer [28] were successfully developed and showed promising results in the reduction of dermal lesions and standard management of acute bleeding in congenital and acquired bleeding disorders. Many groups of researchers, including ours, support the idea that applying EVs as an innovative diagnostic system can be a breakthrough in veterinary medicine such as the application of synthetic polymers in human medicine [29-31].

The use of proteomics to validate biomarkers in EVs in order to facilitate precise diagnosis can be a valuable tool in in the research of parasitic infections [32-34]. Most of proteomic investigations are directed to study milk for use in dairy products and in mastitis, while the investigations in parasitic diseases are still poorly described [35, 36]. The protozoan parasites have developed various strategies to overcome host cell protective mechanisms favoring their survival [37]. Among the strategies used by them, EVs gained attention as an important evasion system during parasite infection. 

The present study reports for the first time the isolation and characterization of EVs from buffalo serum, comparing the EV content from healthy animals, *Theileria* spp*.* positive and *Theileria* spp*.* positive after treatment with dibenzamidine diacetate. Additionally, we were able to identify and quantify possible biomarkers for *Theileria* spp. by using proteomic strategy.

## Methods

### Animals and Biological Samples

Fifty-seven buffaloes of the *Bubalus bubalis* species, 29 males and 28 females of the Murrah breed, of approximately 18 months of age, non-lactating, belonging to a private farm (latitude -22.8744 and longitude -47.9964) were selected as blood donors for this study during the period of September 4, 2014 and March 27, 2017. These animals were bred on pasture supplemented with corn silage (*Zea maiz* L.) and Tifton 85 hay (*Cynodon* spp.), with drinking water and *ad libitum* mineral salt. All buffalo management and health conditions, which include endo and exoparasite vaccination and control schemes, were carried out in accordance with the Brazilian National Animal Health Programs from the Ministry of Agriculture, Livestock and Supply (MAPA). Once anaplasmosis was detected in the buffalo herd, the animals were treated with dibenzamidine diacetate (Ganaseg^®^ Plus, Brazil), according to the manufacturer’s instructions.

Buffaloes were divided into three groups: C - control animals or *Theileria* spp. negative animals; Th - *Theileria* spp. positive animals; ThT - treated animals or *Theileria* spp. negative animals after contact with the parasite and treatment with dibenzamidine diacetate (Ganaseg^^®^^ Plus, Brazil). 

### Sample Collection 

Serum samples were collected from buffaloes (*Bubalus bubalis*) of the Murrah breed from the jugular vein of the animals using sterile needles in tubes without anticoagulant. Serum samples were obtained after centrifuging the tubes at 1,500 *g* for 15 min and stored in a freezer at -80^o^C [24]. 

### Hemoparasite Culture

All blood samples were submitted to culture tubes containing 5 mL of liver Infusion tryptose medium. Blood cultures were kept in an oven at 28-30ºC and after 15 days of inoculation, positive cultures for hemoparasites were confirmed by optical microscopy (1000x magnification). *Theileria* spp. was confirmed in positive cultures by using PCR [38].

### Molecular Analysis

 For DNA extraction, Illustra Blood Genomic Prep Mini Spin Kit (GE Healthcare, USA) was used as recommended by the manufacturer. DNA was quantified using NanoVue (GE Healthcare, USA) and PCR reactions were performed by employing the primers described by Cao et al. [39]. The outer primer, F1 (5′-GAAACGGCTACCACATCT-3′) and R1 (5′-AGTTTCCCCGTGTTGAGT-3′), amplified a 778-bp fragment from the 18S rRNA gene, while the inner primer, F2 (5′-TTAAACCTCTTCCAGAGT-3′) and R2 (5′-TCAGCCTTGCGACCATAC-3′), amplified a 581-bp fragment. The reaction was composed of 10 ng of genomic DNA, 1 µL of each primer (10 (mol/µL), 7.5 µL of ultrapure water and 12.5 µL of the GoTaq^®^ Green Master Mix (Promega, USA). 

Incubation was carried out in a Mastercycler Gradient Thermal Cycler (Eppendorf, Germany). The cycling profile consisted of an initial denaturation at 94ºC for 30 s, followed by 40 cycles of 94ºC for 30 s, annealing at 63.5ºC for 30 s, extension at 72ºC for 1 min and a final extension step at 72ºC for 5 min. The amplicons were visualized in a 1.5% electrophoresis gel stained with SYBR™ Gold nucleic acid gel stain (Life Technologies^^®^^, USA).

### Vesicle Isolation and Purification

For the isolation of vesicles, 750 μL of serum samples were loaded onto Izon qEV size-exclusion chromatography columns pre-equilibrated with PBS buffer (Izon Science, UK) and eluted with PBS buffer according to manufacturer's instructions. The columns contained a resin with a pore size of approximately 75 nm, a bed volume of 10 mL, inner tube diameter of 15.6 mm and 3.0 ± 0.25 mL void volume. Columns were pre-filled with PBS, containing 0.05% sodium azide. After discarding the void volume of the column (3 mL), three fractions of each sample were collected (500 μL each). The protein concentration of each fraction was determined by BCA. 

For EVs characterization, the second and third fractions were submitted to western blotting analysis: 12 μg of proteins were separated on SDS-PAGE 10% [m/v] (VWR^®^ Mini Vertical PAGE System, UK), transferred to a nitrocellulose membrane (OmniPAGE Mini electroblotting system, Cleaver Scientific, UK) [40], and blocked for 1 h at room temperature with blocking buffer (1% [m/v] non-fat milk and 0.2% [m/v] I-block protein-based blocking reagent (Applied Biosystems) dissolved in Tris-buffered saline with 0.1% (v/v) Tween-20 (TBST). Western blotting was performed using the primary antibodies anti-flotillin-1 and anti-CD9 (anti-flotillin-1 ABIN5552770 and anti- bovine CD9 ABIN94242 from Antibodies-online, Aachen, Germany) diluted at 1:500 and raised in goat and mouse, respectively. 

After incubation with the primary antibodies, the membrane was washed three times with TBS-T buffer, and further incubated for 1h at room temperature with horseradish peroxidase-conjugated secondary antibodies for anti-flotilin-1 and raised in mouse for anti-CD9 (diluted at 1:5000, Santa Cruz Biotechnology, Inc.). Membranes were imaged in LI-COR Odissey chemiluminescence and fluorescence imager (LI-COR Biosciences, USA). The bands present on membrane images were quantified by densitometric analysis using freeware ImageJ [41].

### EV Characterization

In order to perform the characterization of isolated EV fractions nanoparticle tracking analysis (NTA) and transmission electron microscopy (TEM) were used. In NTA the EV suspensions were diluted in PBS buffer and analyzed in terms of nanoparticle size using a NS300 NanoSight LM10 (Malvern Instruments GmbH Ltda). Samples were introduced manually into the chamber through sterile syringes, and three videos of 30 s each were captured, with approximately 2000 tracks counted in each measure, at room temperature [42]. For TEM, the EV fractions were diluted 1:10 in PBS, applied onto a 200-mesh carbon-coated grid for 7 min at room temperature, thereafter stained with 1.75% (v/v) uranyl acetate and washed in by sterile H_2_O. The grids were observed using a transmission electron microscope JEM 1400 (Jeol Ltd., Japan) operated at 80 kV [43].

### Proteomic Analysis 

Twelve micrograms of total proteins from samples were diluted to a volume of 50 μL using 0.1 M triethyl ammonium bicarbonate (TEAB, Thermo Scientific, USA), reduced by adding 2.5 μL of 200 mM DTT (60 min, 55°C) (Sigma Aldrich, USA), alkylated by adding 2.5 μL of 375 mM Iodoacetamide (30 min, room temperature in the dark) (Sigma Aldrich, USA) and precipitated with acetone (300 μL) overnight at −20°C. Protein pellets were collected subsequently by centrifugation (8000 *g*, 4°C), dissolved in 50 μL of 0.1 M TEAB and digested using 1 μL of trypsin (1 mg/mL, Promega, USA); trypsin-to-protein ratio 1:35, at 37°C overnight. Tryptic digestion was stopped by acidification, by diluting the digested samples to a 0.5% (v/v) final concentration with formic acid. Peptides were reconstituted and desalted using Waters Sep-pack 50 mg C18 cartridges (Waters MA., USA) performing several washes of 0.1% (v/v) trifluoroacetic acid (TFA). Elution was carried out using 75% (v/v) acetonitrile, 0.1% formic acid and the eluted samples were concentrated using a vacuum concentrator. 

For the EVs proteomic analysis, 1 μg of tryptic peptides was separated on an Ultimate 3500 RSLS nanoflow system (Dionex, Thermo Fisher Scientific, USA) before online ESI-MS/MS analysis in a Q Exactive Plus mass spectrometer (Thermo Fisher Scientific, USA). For EV identification, direct injection onto a PepMap RSLC C18 analytical column (15 cm x 75 μm) with a linear gradient 5-35% mobile phase B [0.1% (v/v) formic acid in 80% (v/v) acetonitrile] over 180 min at a flow rate of 300 nL/min was used to separate peptides. The MS instrument was operated using a Nanospray Flex ion source with a SilicaTipemitter (New Objective, China). The ionization voltage was set to 1.9 kV and the ion transfer capillary temperature to 275°C. MS was operated in positive ion mode using FT HCD MS2 [44]. Full scan FTMS spectra were acquired in range from m/z 380.0 to 1800.0 with a resolution of 60,000. 

Three most intense peaks from MS spectrum were selected for each fragmentation mode. The HCD MS/MS scan was fixed to start from m/z 100 with a resolution of 15000 using MS2 AGC target of 5 x 104. The collision energy was set as 40% NCE and isolation window to ± 1.5 Da. Every precursor ion was repeated twice within a duration time of 30s and was excluded for 20 s. Precursor ions with unassigned charge state, as well as charge states of +1 and more than +7 were excluded from fragmentation. For peptide identification and relative quantification, the SEQUEST algorithm implemented into Proteome Discoverer (version 2.0, Thermo Fisher Scientific, USA) was used. 

Database search against *Bos taurus* FASTA files downloaded from NCBI database (6,347,539 entries) was performed according to the following parameters: two trypsin missed cleavage sites, precursor and fragment mass tolerances of 10 ppm and 0.02 Da, respectively; carbamidomethyl (C) fixed peptide modification, oxidation (M), deamidation (N,Q) dynamic modifications. The false discovery rate (FDR) for peptide identification was calculated using the Percolator algorithm in the Proteome Discoverer workflow based on the search results against a decoy database and was set at 1% FDR. Only proteins with at least two unique peptides were reported as reliable identification.

For the analysis of the proteomic data a matrix compatible with the program MetaboAnalyst 4.0^®^ (https://www.metaboanalyst.ca/) was constructed. The spectral counts were normalized for each protein identified by the weighted average of the triplicates of each sample [45]. Proteins that have been identified in less than 40% of the samples were excluded from the analysis. Principal component analysis (PCA) and partial least squares (PLS) were used as the main method of multivariate analysis. Only signals present in 80% of the samples were considered for the generation of statistical models. 

Proteins were subjected to enrichment analysis for Gene Ontology (GO) terms “molecular function”, “biological process” and “cellular component” using PANTHER software (http://www.pantherdb.org/ - version 13.1). Protein interactions were also investigated in regard to their biological processes using the STRING software (http://string-db.org/ - version 10.5) by using the basic parameters: cut-off score of 0.90, confidence as network edges and PPI enrichment p-value of < 1.0e-16.

## Results

### Theileriosis Diagnosis

**Figure 1.**
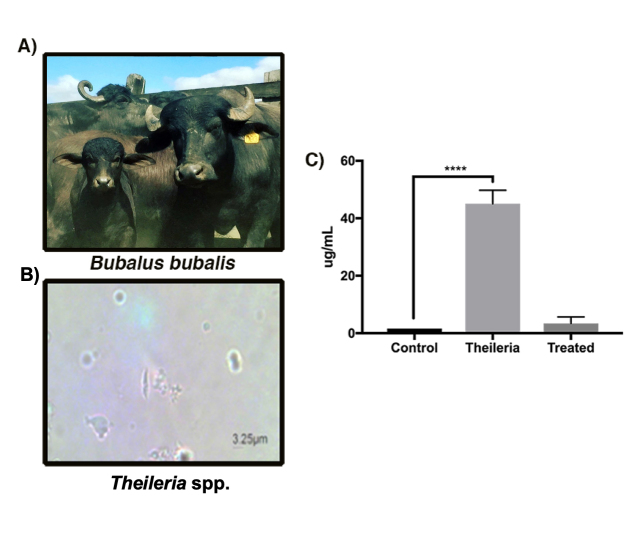
*Theileria* spp. found in infected animals. **(A)** *Bubalus bubalis* buffaloes and **(B)** photomicrography of the *Theileria* spp. parasite. **(C)** DNA quantification showing a reduction of *Theileria* spp. DNA upon dibenzamidine diacetate treatment.

*Theileria* disease is caused by an obligate intracellular parasite, transmitted by a tick vector to the mammalian host (Figure 1A and 1B) [46]. Despite disease diagnosis through the conventional microscopy methodology, PCR detection methods are currently considered as a superior alternative to identify parasite DNA in host blood [47]. Of the 57 animals evaluated, 11 tested positive for *Theileria* spp., four for *Babesia bovis* and five for both parasitic species. PCR assays corroborated the blood culture results. Based on these results, three experimental groups were composed: C - control animals or *Theileria* spp. negative animals (n = 3); Th - *Theileria* spp. positive animals (n = 2) and ThT - treated animals or *Theileria* spp. negative animals after contact with parasite and treated with dibenzamidine diacetate (Ganaseg^®^ Plus) (n = 3). 

DNA quantification in serum samples indicated a concentration of more than 40 µg/mL in *Theileria* spp*.* positive animals and its concentration near of 0 µg/mL in control animals. Animals treated during three months with dibenzamidine diacetate showed a significant decrease in DNA amount, revealing the efficiency of treatment (Figure 1C).

### Isolation and Characterization of EVs

**Figure 2.**
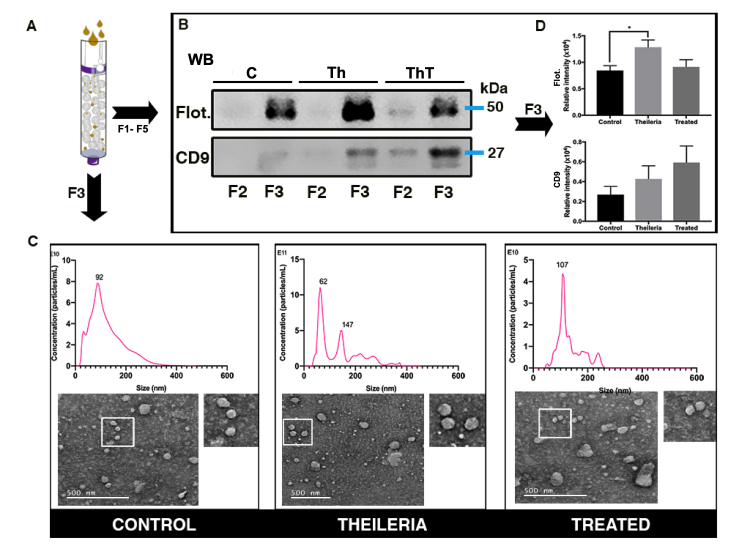
Isolation and characterization of EVs from buffalo serum. **(A)** Izon qEV size-exclusion chromatography columns. **(B)** Western blotting of Fractions 2 (F2) and 3 (F3) to the EV markers flotillin-1 and CD9 in control animals, *Theileria* spp. positive animals and treated animals. **(C)** Nanoparticle tracking analysis and transmission electron microscopy analysis in Fraction 3. **(D)** Densitometry of WB bands of Fraction F3.

Given the confirmation of presence and/or absence of *Theileria* DNA, we further evaluated the serum samples from buffaloes regarding the presence of EVs. The serum was centrifuged at 4200 ×*g* for 10 min at 22C. Serum from the three experimental animal groups were fractionated by Izon qEV size-exclusion chromatography columns (Izon Science, UK) (Figure 2A), resulting in five fractions of 500 (L (F1 to F5). All fractions were analyzed by western blotting (data not shown) and fractions F2 and F3 showed signals corresponding to the specific EV markers: flotillin-1 (cytosolic protein recovered on EVs) and CD9 (transmembrane protein associated with endosomes). Western blotting (WB) analyses have confirmed the efficiency of EV purification (Figure 2B). The F3 fractions from control animals (C), *Theileria* spp. positive animals (Th) and treated animals (ThT) were submitted to nanoparticle tracking and transmission electron microscopy analysis (Figure 2C). The vesicles present in fractions had sizes ranging from 140.2 nm for control animals, 124.0 nm for *Theileria* positive animals (Th) to 138.8 nm for treated animals (ThT), which is compatible with EV sizes, confirming the quality of purified vesicles (Table 1).

**Table 1.** Nanoparticle tracking analysis of extracellular vesicles obtained from *Bubalus bubalis* sera found in F3 fractions isolated from control animals, *Theileria* spp. positive animals and treated animals (± standard error). Values are represented in nanometers (nm).

		Read 1	Read 2	Read 3	Average	Standard deviation
Control animals	Mean	154.1	133.5	133.0	140.2	6.9
	Mode	67.0	58.1	42.9	56.0	7.0
	SD	97.2	86.6	82.1	88.6	4.5
	D10	58.4	50.2	38.7	49.1	5.7
	D50	133.6	103.4	130.1	122.4	9.5
	D90	279.6	259.9	235.1	258.2	12.9
***Theileria* spp. positive**	Mean	125.6	116.4	130.0	124.0	4.0
	Mode	88.5	29.0	78.3	65.3	18.4
	SD	71.9	70.5	63.6	68.7	2.6
	D10	42.9	30.3	63.6	45.6	9.7
	D50	93.4	97.6	110.1	100.3	5.0
	D90	217.1	209.0	223.1	216.4	4.1
**Treated animals**	Mean	149.9	138.0	128.6	138.8	6.2
	Mode	132.5	91.1	109.3	111.0	12.0
	SD	52.1	50.0	41.7	47.9	3.2
	D10	102.4	82.7	78.8	88.0	7.3
	D50	128.4	121.3	106.2	118.6	6.5
	D90	232.1	226.0	190.4	216.2	13.0

### Detection of Flotillin and CD9 in EVs

Flotillin is a cytosolic protein that is found in EVs due to its ability to bind to membranes. This protein is considered a canonical marker for EVs. We have detected flotillin-1 on fractions F2 and F3 obtained from EV purification in the control animals (C), *Theileria* spp. positive animals (Th) and treated animals (ThT) (Figure 2B). To determine whether the amount of this protein varied among the groups, we quantified WB bands by densitometry, which revealed that flotillin-1 increased significantly in *Theileria* spp. positive animals when compared with controls. Interestingly, after treatment with dibenzamidine diacetate, the levels of this marker decreased in treated animals, being comparable with the control animals, as can be seen in Figure 2D. The CD9 tetraspanin membrane protein is another EV marker that was detected in F2 and F3 samples. This protein was also increased in *Theileria* spp. positive animals. However, its levels did not decrease upon treatment with dibenzamidine diacetate; on the other hand, they increased in treated animals, as shown in Figure 2D.

### Proteomic Analysis

**Figure 3.**
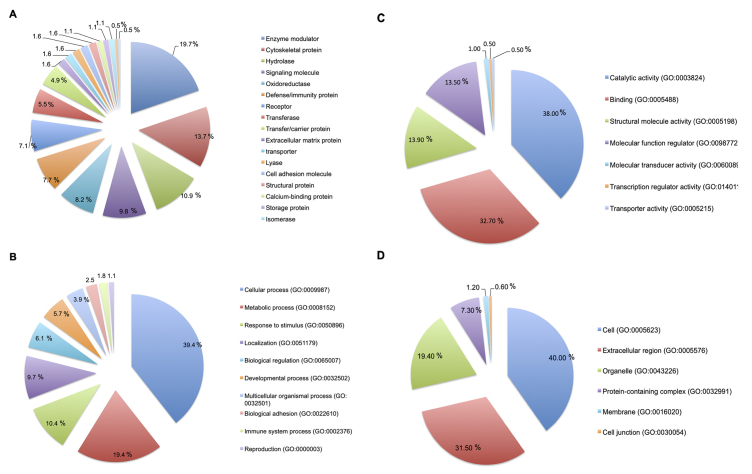
Gene Ontology enrichment analysis of the 306 proteins identified in EVs from buffalo serum. **(A)** Protein classes, **(B)** biological process, **(C)** molecular function and **(D)** cellular component.

**Figure 4.**
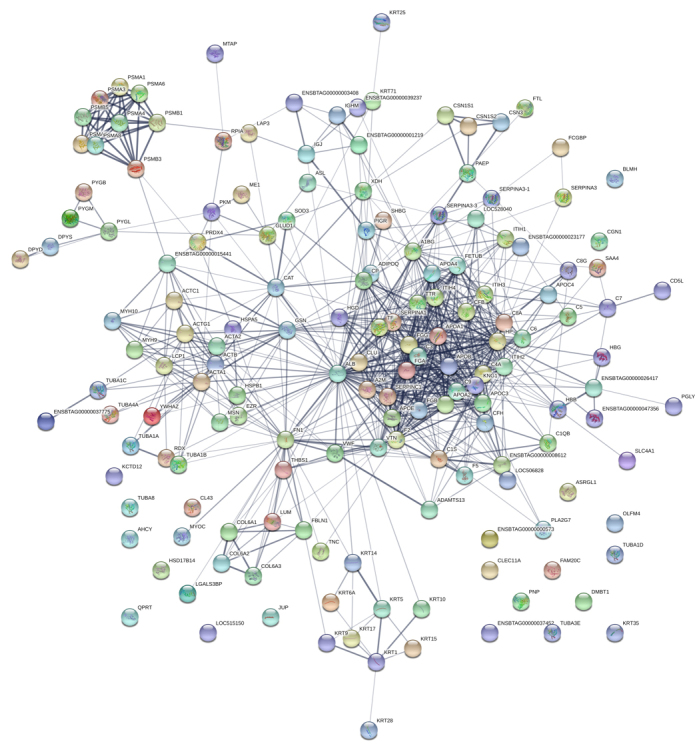
Interaction network among major proteins identified in EV from buffalo serum. The circles represent proteins while the straight lines represent the interactions among different proteins. The stronger associations are represented by thicker lines.

**Figure 5.**
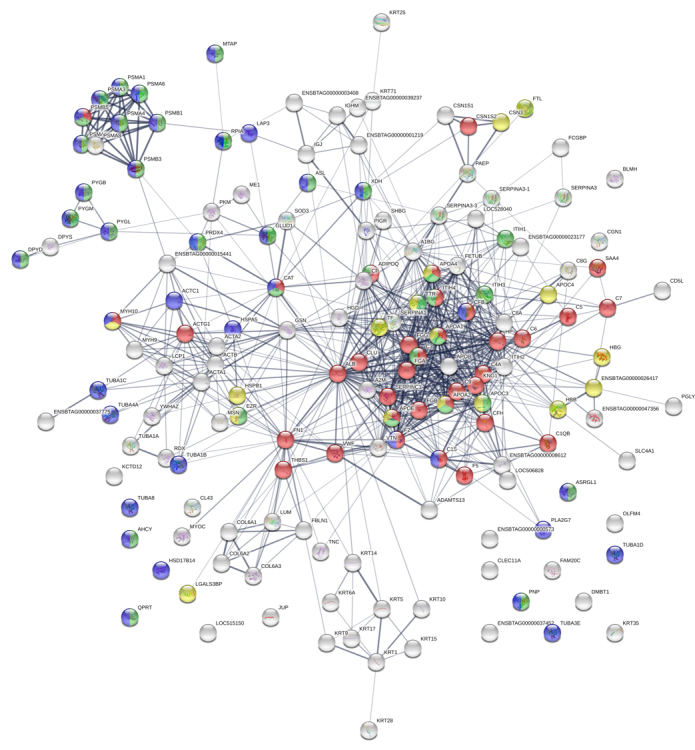
Main biological processes and molecular functions: dark green circles indicate cellular metabolic processes; yellow circles show transport; purple ones designate catalytic activity, light green circles mark binding whereas red ones represent the proteins involved in the response to stress.

**Figure 6.**
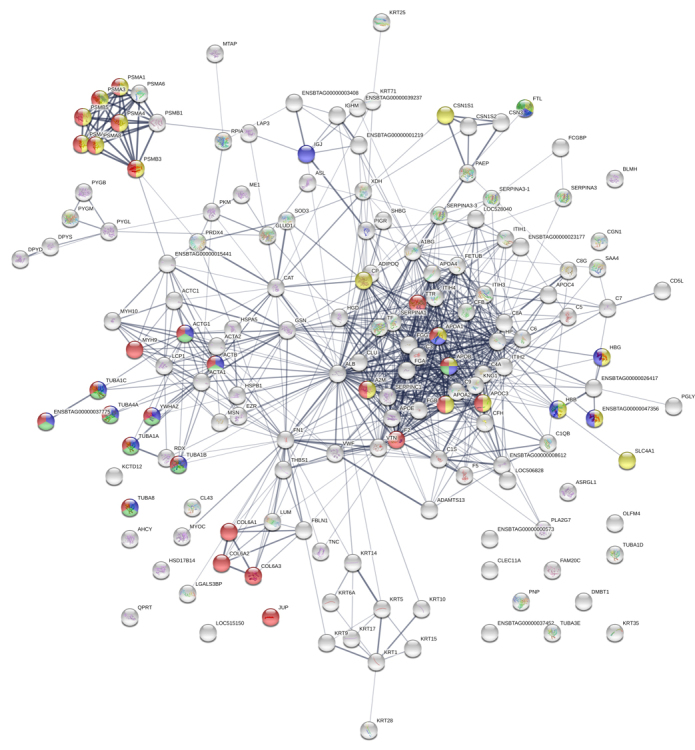
Principal Reactome pathways: yellow circles indicate the proteins involved in transport of small molecules; green circles represent membrane trafficking; purple ones show vesicle-mediated transport and red circles depict proteins involved in signal transduction. The protein network was analyzed using STRING software and the associations between proteins were detected using the Kyoto Encyclopedia of Genes and Genomes (KEGG) tool.

A total of 306 proteins were identified and analyzed in fraction F3 of controls, *Theileria* spp. positive and treated animals. Such proteins are listed in the Additional files 1, 2 and 3. Gene ontology (GO) enrichment analysis have evidenced 217 genes involved in the protein expression, in which 19 protein classes can be distinguished: enzyme modulator, cytoskeletal protein, hydrolase, signalling molecule, oxidoreductase, defense protein, receptor, transferase, carrier protein, extracellular matrix protein, transport, lyase, cell adhesion molecule, structural protein, calcium-binding protein, storage protein, isomerase, chaperone and surfactant (Figure 3A). GO annotations “biological process”, “molecular function” and “cellular component” are shown in the Figures 3B, 3C and 3D, respectively. Additionally, the protein-protein interaction map provided a better understanding of the relationship among the proteins present in extracellular vesicles from buffalo serum (Figure 4 and Figure 5). The major Reactome pathways can be distinguished into membrane trafficking of small molecules; vesicle-mediated transport and signal transduction (Figure 6).

PCA and PLS analysis (Additional file 4) of the samples revealed statistical differences between control animals and *Theileria* spp. positive buffaloes whereas treated animals were considered similar to the control animals. It is possible to observe that only PCA was not sufficient to distinguish *Theileria* positive from control animals. Conversely, the PLS analysis satisfactorily divided the groups. Five proteins showed differential expression between controls and *Theileria* positive animals (Additional files 5 and 6): creatine kinase U-type (code access: Q9TTK8), L-lactate dehydrogenase A chain (code access: P19858), L-lactate dehydrogenase (code access: B0JYN3), L-lactate dehydrogenase (code access: Q5E9B1), and betaine-homocysteine S-methyltransferase 1 (code access: Q5I597). The ESI-MS/MS data were deposited in the PeptideAtlas repository (http://www.peptideatlas.org/PASS/PASS01243) with the dataset identifier PASS01243.

## Discussion

Extracellular vesicles (EVs) are spontaneously released from cells, but their production may be higher or lower depending on the infection. They have been increasingly studied for their mechanism of intercellular communication and may be of particular significance in infectious diseases. EVs can potentially benefit the host immune response or promote alternative pathways for pathogen survival [48]. The present results corroborate previous findings describing EVs as players in parasite-host interactions [48,49]. We are aware of the challenges of studying EVs and, in this aspect, we emphasize that one of the limitations of the present study, also reported in the literature, refers to the low number of samples due to the high costs of analysis. 

Despite the knowledge about differences in their biogenesis and function, differentiation between exosomes and EVs is not always possible, so that a consensus and establishment of standardized methods is necessary to provide more reliable and interchangeable premises among studies [50-52].

The apicomplexan phylum is composed of more than 6,000 named species, including genera of clinical and/or economic importance in human and veterinary medicine. Apicomplexans cause human malaria (*Plasmodium* spp.)*,* theileriosis (*Theileria* spp.), coccidiosis (*Eimeria* spp.), cryptosporidiosis (*Cryptosporidium* spp.) and toxoplasmosis (*Toxoplasma gondii*). In fact, all animal species are believed to serve as host to at least one Apicomplexan species [53]. Theileriosis is a tick-borne disease that has historically caused great economic losses in the farming industry. *Theileria* species causes many diseases in livestock: corridor disease (*T. parva*), East Coast fever (*T. parva*), tropical theileriosis (*T. annulata*) in cattle and malignant theileriosis (*T. lestoquardi*) in goats and sheep [54]. Affections caused by apicomplexan parasites are also considered emerging zoonoses [11,13,15,55,56]. 

This communication evidenced the isolation and enrichment of EVs in *Theileria* infections in buffaloes, confirming the literature data about EVs in parasitic infections [57]. Isolation of vesicles was conducted by means of size exclusion chromatography and identification of EVs was assessed through western blotting by the presence of tetraspanin CD9 and membrane lipids raft-associated Flotllin-1 [4]. These markers are compatible with validated studies of exosomes; however, they are not specific for exosomes and could represent other types of EVs, thus being referred to as exosome-like due to the presence of microvesicles after size-exclusion [58].

These results remarkably suggest that for exosome-like vesicles isolation using size exclusion chromatography columns, the third fraction (F3) contain the higher number of vesicles, and should be assessed for further analysis, in agreement with the literature [58]. Despite the fact that ultracentrifugation (UC) remains the most frequently used primary isolation technique for EVs, vesicles isolated by UC are known to suffer from non-vesicular macromolecule contamination and vesicular aggregation, hampering omics and functional analysis. For this reason, UC is often combined with other purification techniques, which implies in more time-consuming protocols [58,59]. The use of size-exclusion chromatography and other chromatographic techniques (e.g. HPLC) was recently validated and it is increasingly used to isolate Evs, since it provides lower contamination with macromolecular complexes than ultracentrifugation. Nevertheless, different isolation techniques might isolate different EV subpopulations, so that, identifying surface markers of a certain subpopulation would be required to achieve the highest purity, such as affinity-based isolations [60].

Our results showed an increase in flotillin quantification in EVs of *Theileria* spp. positive group in comparison to control, and a decrease of this protein as a result of a treatment with the drug Ganaseg^®^ Plus. Although we understand the necessity of further investigation about the relationship of EVs with *Theileria* infections, these results can be considered as a first evidence on the contribution of EVs in parasitic diseases, and more than that, suggest that the amount of EVs is positively related with the degree of infection. 

This hypothesis is supported by the literature data demonstrating the increase of exosomes followed by parasite load increases. This can be explained by the assumption that exosomes carry genes responsible for essential mechanisms for the disease development (parasite-host) such as sustain itself for a longer time, increasing consecutively the infection time [52]. Over the past three decades, many proteomics studies performed on EVs have elucidated their diverse roles exerted by theses vesicles. 

The protein-protein interaction analysis from buffalo blood vesicles has evidenced biological and molecular processes associated with binding, regulation of metabolic processes, transport, catalytic activity and response to stress. Besides, it was possible to Reactome pathways as signal transduction, transport of small molecules, membrane trafficking and vesicle-mediated transport.

The PLS results suggest that extracellular vesicles from affected animals can be distinguished from those of healthy ones by presenting lactic acid that is related to the reduced use of pyruvate in the tricarboxylic acid cycle and the increase of anaerobic glycolysis [61-62]. Lactate may also result from the activated lactate dehydrogenase, which results in increased oxidative stress [63-65]. Creatine was also markedly increased in unhealthy animals, leading to adverse changes and serious damage to renal function [66]. Since this compound is thought to exert direct antioxidant effects [67,68], its increase may reflect a protective mechanism against the enhanced oxidation [69,70], corroborating the data obtained in the Gene Ontology enrichment analysis and protein-protein interaction network shown in Figures 3 and 4. There are few descriptions about these pathways and their role in EVs derived from infected environments. Additional studies should be done over the years conducting to better elucidations about the extracellular vesicle pathways in protozoan parasites. 

Based on the described results, we suggest that *Theileria* parasites may use EVs as important pawns in their infection processes. The real role of these vesicles is still unclear; however, their presence, as well as their increase in blood from unhealthy animals highlight a possible new approach to understand parasite-host relationship. Specifically, for *Theileria* spp. infections, given the importance of buffalos for the production of human and veterinary health products, we can assume that our data strongly contribute to a future development of an improved diagnostic tool that could bring economic and public health advantages. 

## Conclusion

For the first time EVs from buffalo blood infected with *Theileria* spp. were successfully isolated and characterized. This is an advance in the knowledge of host-parasite relationships contributing to understanding the evasion mechanisms of the parasite. These findings may pave the way for the search of new EV candidate-markers aiming to a better management of buffaloes to produce safe biologicals. Further steps are strongly encouraged in relation to expanded EVs studies in Apicomplexa infections in animals.

### Abbreviations

EVs: extracellular vesicles; FDR: false discovery rate; GO: Gene Ontology; HPLC: high-performance liquid chromatography; LEVs: large extracellular vesicles; MVBs: multivesicular bodies; MVEs: multivesicular endosomes; NTA: nanoparticle tracking analysis; PBS: phosphate buffered saline; PCA: principal component analysis; PCR: polymerase chain reaction; PLS: partial least squares; SEVs: small extracellular vesicles; TBST: tris-buffered saline and polysorbate 20 buffer; TEM: transmission electron microscopy; UC: ultracentrifugation; WB: western blotting.

## Supplementary material

The following online material is available for this article

Additional file 1. Summary of extracellular vesicle proteins of *Theileria* spp. isolated from serum of positive buffaloes present in Fraction F3.

Additional file 2. Summary of extracellular vesicle proteins isolated from serum of control animals present in Fraction F3.

Additional file 3. Summary of extracellular vesicle proteins isolated from serum of treated animals present in Fraction F3.

Additional file 4. Analysis of proteomic data in which control animals and *Theileria* spp. positive animals presented relevant statistical differences. **(A)** Principal component analysis (PCA). **(B)** Partial least squares (PSL).

Additional file 5. Proteins identified with differential expression by fold change analysis.

Additional file 6. Proteins identified with differential expression by t-tests.
