# Supplementary material for: Extracellular vesicles in infectious diseases caused by protozoan parasites in buffaloes
Source: J Venom Anim Toxins Incl Trop Dis. 2020 May 29;26:e20190067. doi: 10.1590/1678-9199-JVATITD-2019-0067 (PMC7262785; doi:10.1590/1678-9199-JVATITD-2019-0067)
Supplement: Additional file 1. [file 1678-9199-jvatitd-26-e20190067-s1.pdf]

*J Venom Anim Toxins incl Trop Dis*

https://doi.org/10.1590/1678-9199-JVATITD-2019-0067

# Supplementary Material to "Extracellular vesicles in infectious diseases caused by protozoan parasites in buffaloes"

**Additional file 1.** Summary of extracellular vesicle proteins of *Theileria* spp. isolated from serum of positive buffaloes present in Fraction F3.

| Code access | Description* | MW [kDa] | Coverage | Unique peptides | Score |
|---|---|---|---|---|---|
| **E1BNR0** | Apolipoprotein | 515.438 | 22.92533 | 89 | 371.6080978 |
| **Q2UVX4** | Complement C3 | 187.135 | 38.83203 | 61 | 435.1392704 |
| **A0A0F6QNP7** | Complement component 3 | 187.064 | 38.4106 | 60 | 432.9851233 |
| **G3X7A5** | Complement C3 | 187.028 | 37.86875 | 60 | 422.0706013 |
| **B8Y9S9** | Fibronectin 1 | 262.263 | 21.91035 | 33 | 124.5409169 |
| **G5E5A9** | Fibronectin | 271.952 | 20.17756 | 32 | 120.5719106 |
| **P07589** | Fibronectin | 271.983 | 20.17756 | 32 | 120.5719106 |
| **B8Y9T0** | Fibronectin 1 | 248.974 | 21.11993 | 31 | 117.9743552 |
| **P15497** | Apolipoprotein A-I | 30.258 | 61.88679 | 23 | 155.5061482 |
| **Q0VCM4** | Glycogen phosphorylase | 97.394 | 22.56169 | 19 | 42.05386758 |
| **F1MI18** | Uncharacterized protein | 165.654 | 14.81732 | 18 | 61.36519742 |
| **F1MJK3** | Uncharacterized protein | 165.506 | 14.7973 | 18 | 61.36519742 |
| **A0A140T897** | Serum albumin | 69.278 | 29.98353 | 17 | 64.88931954 |
| **P02769** | Serum albumin | 69.248 | 29.98353 | 17 | 64.88931954 |
| **P23805** | Conglutinin | 37.971 | 21.56334 | 17 | 227.0630013 |
| **P80457** | Xanthine dehydrogenase/oxidase | 146.696 | 12.98799 | 16 | 34.40082884 |
| **F1MUT3** | Xanthine dehydrogenase/oxidase | 146.669 | 12.98799 | 16 | 34.40082884 |
| **B0JYQ0** | ALB protein | 69.248 | 25.86491 | 15 | 57.46882677 |
| **F1MVP0** | ADAM metallopeptidase with thrombospondin type 1 | 151.374 | 13.80042 | 15 | 63.02905428 |
| **F1N0R5** | von Willebrand factor | 307.475 | 6.552707 | 14 | 48.45272136 |
| **F1MAV0** | Fibrinogen beta chain | 56.405 | 36.36364 | 14 | 51.43162334 |
| **E1BH06** | Uncharacterized protein | 192.644 | 21.65422 | 14 | 121.2117517 |
| **F5XVA9** | von Willebrand factor | 307.745 | 6.548043 | 14 | 48.45272136 |

| Code access | Description* | MW [kDa] | Coverage | Unique peptides | Score |
|---|---|---|---|---|---|
| **P02676** | Fibrinogen beta chain | 53.306 | 33.76068 | 13 | 46.7038182 |
| **F1MY85** | Complement C5a anaphylatoxin | 188.927 | 9.660107 | 13 | 35.12020373 |
| **V6F869** | Apolipoprotein A-I-like | 23.629 | 51.44231 | 13 | 80.3775357 |
| **A0A0F6QMJ3** | Complement component 5 | 188.691 | 9.660107 | 13 | 35.12020373 |
| **F1N3Q7** | Apolipoprotein A-IV | 42.963 | 29.47368 | 12 | 51.3780371 |
| **Q32PJ2** | Apolipoprotein A-IV | 42.991 | 29.47368 | 12 | 51.3780371 |
| **G3N0I4** | Cytosol aminopeptidas | 54.047 | 34.73896 | 12 | 45.8811245 |
| **P00727** | Cytosol aminopeptidas | 56.254 | 33.33333 | 12 | 45.8811245 |
| **Q693V9** | Complement component 3d | 34.422 | 40.26403 | 11 | 77.3899281 |
| **Q3MHL4** | Adenosylhomocysteinase 3 | 47.607 | 24.30556 | 10 | 32.89724624 |
| **P00432** | Catalase | 59.878 | 21.44213 | 10 | 34.88407671 |
| **Q7SIH1** | Alpha-2-macroglobulin | 167.47 | 22.45033 | 10 | 146.282555 |
| **Q95KV5** | Fibronectin | 38.32 | 42.07493 | 10 | 37.45227361 |
| **F1MQ37** | Myosin heavy chain 9 | 226.962 | 6.364562 | 10 | 19.91305554 |
| **F1MD73** | Uncharacterized protein | 189.978 | 16.90463 | 10 | 109.3602887 |
| **A5PJE3** | Fibrinogen alpha chain | 66.957 | 14.79675 | 10 | 49.72597051 |
| **P02672** | Fibrinogen alpha chain | 66.971 | 14.79675 | 10 | 49.72597051 |
| **A5D9E9** | Complement C1r subcomponent precursor | 80.161 | 17.44681 | 9 | 32.06269646 |
| **Q03247** | Apolipoprotein | 35.958 | 26.58228 | 9 | 85.04481769 |
| **Q2TBU0** | Haptoglobin | 44.831 | 22.69327 | 9 | 26.58675003 |
| **E1BI98** | Collagen type VI alpha 1 chain | 108.603 | 10.32132 | 9 | 20.81394196 |
| **G5E513** | Uncharacterized protein | 49.939 | 45.95186 | 9 | 540.2513834 |
| **A6QPX7** | FGB protein (Fragment) | 37.907 | 37.57576 | 9 | 30.26272595 |
| **P01030** | Complement C4 | 101.817 | 17.06522 | 9 | 43.73454607 |
| **E1B805** | Uncharacterized protein | 187.149 | 6.09319 | 9 | 16.55716145 |
| **G3X6K8** | Haptoglobin | 44.845 | 22.69327 | 9 | 26.58675003 |
| **A0A140T881** | Apolipoprotein | 36.017 | 26.58228 | 9 | 85.04481769 |
| **G3N3D4** | Potassium channel tetramerization domain containing 12 | 35.701 | 28.52761 | 9 | 38.9555769 |
| **A7YWR0** | Apolipoprotein | 36.051 | 26.58228 | 9 | 85.04481769 |
| **E1BFN6** | Dihydropyrimidinase | 56.267 | 18.60465 | 9 | 32.63112199 |
| **P81187** | Complement factor B | 85.312 | 14.32326 | 8 | 22.24805796 |
| **P17697** | Clusterin | 51.081 | 13.66743 | 8 | 32.22156239 |
| **F1N076** | Ceruloplasmin | 123.731 | 12.53456 | 8 | 19.07722878 |

| Code access | Description* | MW [kDa] | Coverage | Unique peptides | Score |
|---|---|---|---|---|---|
| **R9QSM8** | Alpha-2-macroglobulin | 133.302 | 25.95993 | 8 | 131.0675713 |
| **F1N514** | CD5 antigen-like precursor | 50.305 | 15.89404 | 7 | 86.63707793 |
| **Q3SZZ9** | FGG protein | 49.136 | 18.3908 | 7 | 32.44846714 |
| **Q0VCX1** | Complement C1s subcomponent | 76.56 | 8.853411 | 7 | 23.25133181 |
| **F1MJ12** | Complement C1s subcomponent | 77.332 | 8.776978 | 7 | 23.25133181 |
| **F1MGU7** | Fibrinogen gamma-B chain | 50.2 | 18.05869 | 7 | 32.44846714 |
| **Q95KV4** | Fibronectin (Fragment) | 35.464 | 35.9375 | 7 | 25.80675912 |
| **Q3Y5Z3** | Adiponectin | 26.117 | 32.91667 | 7 | 28.58874869 |
| **Q0ZCB4** | Apolipoprotein | 27.057 | 29.95781 | 7 | 64.91904032 |
| **A2VDY5** | Hydroxysteroid (17-beta) | 28.378 | 37.40741 | 7 | 19.28046763 |
| **Q9MYP6** | 17-beta-hydroxysteroid dehydrogenas | 28.404 | 37.40741 | 7 | 19.28046763 |
| **A6QNW7** | CD5L protein | 50.179 | 15.96452 | 7 | 86.63707793 |
| **Q5E9B1** | L-lactate dehydrogenase | 36.7 | 18.56287 | 6 | 8.872087121 |
| **B7FEK7** | 43kDa collectin | 33.594 | 10.28037 | 6 | 82.2378906 |
| **B0JYN3** | L-lactate dehydrogenase | 36.701 | 18.56287 | 6 | 8.872087121 |
| **Q28178** | Thrombospondin-1 | 129.451 | 6.495726 | 6 | 9.785889983 |
| **A7E3W2** | Galectin-3-binding protein | 62.087 | 12.07207 | 6 | 24.50829315 |
| **F1MKG2** | Collagen type VI alpha 2 chain | 109.253 | 7.594937 | 6 | 13.22950125 |
| **B8Y898** | Malic enzyme | 63.853 | 16.11208 | 6 | 21.34723735 |
| **P02663** | Alpha-S2-casein | 26.002 | 27.02703 | 6 | 13.52538526 |
| **F1MC11** | Keratin, type I cytoskeletal 14 | 51.879 | 15.72327 | 6 | 28.12601042 |
| **A6QNZ7** | Keratin 10 | 54.816 | 9.695817 | 6 | 23.54977572 |
| **P12799** | Fibrinogen gamma-B chain | 50.212 | 14.63964 | 6 | 26.34777749 |
| **Q3SYR8** | Immunoglobulin J chain | 17.846 | 22.92994 | 6 | 61.62939811 |
| **F1N3A1** | Thrombospondin-1 | 129.309 | 6.495726 | 6 | 9.785889983 |
| **A7E3D5** | Proteasome subunit alpha type (Fragment) | 26.551 | 31.22363 | 6 | 18.23925734 |
| **K4JR81** | Alpha-2-macroglobulin variant 12 | 61.673 | 12.02873 | 6 | 35.65589309 |
| **Q3ZBG0** | Proteasome subunit alpha type-7 | 27.852 | 29.83871 | 6 | 18.23925734 |
| **F1MSZ6** | Antithrombin-III OS=Bos taurus | 52.407 | 16.98925 | 6 | 26.68186378 |
| **P00735** | Prothrombin | 70.461 | 12.32 | 6 | 12.0139544 |
| **F1MFY6** | Collectin-43 precursor | 24.905 | 13.46939 | 6 | 82.2378906 |
| **Q58D62** | Fetuin-B | 42.636 | 13.95349 | 6 | 9.815567017 |
| **P06394** | Keratin, type I cytoskeletal 10 | 54.815 | 9.695817 | 6 | 23.54977572 |

| Code access | Description* | MW [kDa] | Coverage | Unique peptides | Score |
|---|---|---|---|---|---|
| **Q3SZJ0** | Argininosuccinate lyase | 52.71 | 15.4334 | 5 | 13.05015182 |
| **G5E5C3** | Proteasome subunit alpha type | 27.267 | 20 | 5 | 10.13151824 |
| **F1MM86** | Complement component C6 | 104.455 | 4.828326 | 5 | 9.712654352 |
| **G3N0V2** | Keratin 1 | 63.113 | 8.085809 | 5 | 38.03936386 |
| **O02808** | Von Willebrand factor (Fragment) | 59.57 | 14.7541 | 5 | 21.56231964 |
| **Q3SYT3** | Complement C1r subcomponent precursor | 44.528 | 17.51269 | 5 | 19.45690751 |
| **Q2YDE4** | Proteasome subunit alpha type-6 | 27.382 | 19.9187 | 5 | 10.13151824 |
| **F1MTV7** | Argininosuccinate lyase | 52.682 | 15.4334 | 5 | 13.05015182 |
| **B8YB77** | Malic enzyme | 63.332 | 13.55634 | 5 | 18.47211838 |
| **G3X6N3** | Serotransferrin | 77.616 | 7.386364 | 5 | 9.215728402 |
| **Q5I597** | Homocysteine | 44.85 | 15.23342 | 5 | 16.67927086 |
| **F1MRZ6** | Tenascin C | 244.461 | 3.272075 | 5 | 9.360537648 |
| **G5E5T5** | Uncharacterized protein | 42.442 | 54.24165 | 5 | 514.9136553 |
| **Q3SYR5** | Apolipoprotein C-IV | 14.428 | 48.8189 | 5 | 32.75496709 |
| **Q29RU4** | Complement component C6 | 104.473 | 4.828326 | 5 | 9.712654352 |
| **Q3T063** | Nicotinate-nucleotide pyrophosphorylase | 31.131 | 20.40134 | 5 | 16.96804833 |
| **E1BL29** | Bleomycin hydrolase | 52.896 | 19.38998 | 5 | 23.60210347 |
| **Q29443** | Serotransferrin | 77.703 | 7.386364 | 5 | 9.215728402 |
| **F1MHB8** | Nicotinate-nucleotide pyrophosphorylase | 31.165 | 20.40134 | 5 | 16.96804833 |
| **P41361** | Antithrombin-III | 52.314 | 14.83871 | 5 | 20.79067826 |
| **Q71U44** | Fibronectin (Fragment) | 45.802 | 17.96117 | 5 | 26.43597722 |
| **A0JN60** | Tenascin C | 190.961 | 4.180985 | 5 | 9.360537648 |
| **E1BH94** | Peptidoglycan recognition protein 2 | 59.793 | 14.56835 | 5 | 21.18341041 |
| **F1MRZ5** | Tenascin C | 191.032 | 4.180985 | 5 | 9.360537648 |
| **Q2KIV9** | Complement C1q subcomponent subunit B | 26.383 | 17.81377 | 4 | 17.81737649 |
| **Q28194** | Thrombospondin-1 | 25 | 21.83406 | 4 | 9.785889983 |
| **Q9TUQ0** | Anion exchange protein | 95.582 | 4.561404 | 4 | 9.179233432 |
| **Q9TTK8** | Creatine (phospho) kinase | 46.867 | 15.86538 | 4 | 13.18331146 |
| **Q32KL2** | Proteasome subunit beta type-5 | 28.591 | 20.91255 | 4 | 8.623464584 |
| **P63258** | Actin, cytoplasmic 2 | 41.766 | 14.66667 | 4 | 12.56157732 |
| **B5B3R8** | Alpha S1 casein | 24.427 | 19.15888 | 4 | 23.83278966 |
| **A0A1B0Z542** | Heat shock 27 kDa protein 1 | 22.231 | 15.9204 | 4 | 8.032542706 |
| **Q9XSW5** | Anion exchange protein | 104.308 | 4.193548 | 4 | 9.179233432 |

| Code access | Description* | MW [kDa] | Coverage | Unique peptides | Score |
|---|---|---|---|---|---|
| **F1MNW4** | Inter-alpha-trypsin inhibitor heavy chain H2 | 106.09 | 6.236786 | 4 | 11.40782952 |
| **Q3MHF7** | S-methyl-5'-thioadenosine phosphorylase | 31.236 | 18.0212 | 4 | 8.752094269 |
| **F1N3V0** | Malic enzyme | 63.746 | 10.36907 | 4 | 16.47507405 |
| **F1MYN5** | Fibulin-1 | 77.434 | 9.490085 | 4 | 13.97474813 |
| **A5D7S8** | Fibulin-1 | 77.478 | 9.490085 | 4 | 13.97474813 |
| **A0A1C9EIX3** | Heat shock protein family B member 1 variant 1 | 22.351 | 15.9204 | 4 | 8.032542706 |
| **Q58DP7** | Heat shock 27kDa protein 1 | 17.545 | 20.64516 | 4 | 8.032542706 |
| **E9RHW1** | Heat shock 27kDa protein 1 | 22.379 | 15.9204 | 4 | 8.032542706 |
| **Q3T0X5** | Proteasome subunit alpha type-1 | 29.567 | 16.73004 | 4 | 15.45982051 |
| **P02662** | Alpha-S1-casein | 24.513 | 19.15888 | 4 | 23.83278966 |
| **P60712** | Actin, cytoplasmic 1 | 41.71 | 14.66667 | 4 | 12.56157732 |
| **P31976** | Ezrin | 68.717 | 5.851979 | 4 | 6.883066654 |
| **Q3ZCK9** | Proteasome subunit alpha type-4 | 29.465 | 15.32567 | 4 | 9.53579998 |
| **Q9TUQ1** | Anion exchange protein | 74.3 | 5.882353 | 4 | 9.179233432 |
| **A0A1C9EIX6** | Heat shock protein family B member 1 variant 2 | 22.389 | 15.9204 | 4 | 8.032542706 |
| **A5D984** | Pyruvate kinase | 57.912 | 12.05273 | 4 | 12.27132821 |
| **Q28085** | Complement factor H | 140.282 | 3.964401 | 4 | 7.379358768 |
| **F1MC45** | Complement factor H precursor | 96.53 | 5.875441 | 4 | 7.059792042 |
| **F1MRD0** | Actin, cytoplasmic 1 | 41.825 | 14.66667 | 4 | 12.56157732 |
| **Q9BGI2** | Peroxiredoxin-4 | 30.722 | 25.18248 | 4 | 19.38334894 |
| **Q3ZC87** | Pyruvate kinase (Fragment) | 61.389 | 11.32743 | 4 | 12.27132821 |
| **E1BEL7** | Heat shock protein beta-1 | 22.564 | 15.76355 | 4 | 8.032542706 |
| **Q7M2T6** | Band 3 anion transport protein (Fragments) | 34.3 | 12.7451 | 4 | 9.179233432 |
| **A5D7R6** | ITIH2 protein | 106.12 | 6.236786 | 4 | 11.40782952 |
| **G3X7S2** | Heat shock protein beta-1 | 17.432 | 20.77922 | 4 | 8.032542706 |
| **Q1JQB0** | Collagen type VI alpha 2 chain | 97.075 | 4.907306 | 4 | 7.107832909 |
| **Q6T182** | Sex hormone-binding globulin (Fragment) | 40.069 | 13.74663 | 3 | 8.049062967 |
| **Q29RQ1** | Complement component C7 | 93.029 | 3.677343 | 3 | 6.569371939 |
| **G5E5H7** | Uncharacterized protein | 19.898 | 21.34831 | 3 | 5.753308058 |
| **K4JF16** | Alpha-2-macroglobulin variant 23 | 101.296 | 25.77434 | 3 | 99.956056 |
| **Q2HJ86** | Tubulin alpha-1D chain | 50.251 | 9.292035 | 3 | 7.207318544 |
| **P81947** | Tubulin alpha-1B chain | 50.12 | 9.312639 | 3 | 7.207318544 |
| **A3KLR9** | Superoxide dismutase [Cu-Zn] | 26.16 | 16.59751 | 3 | 11.58625424 |

| Code access | Description* | MW [kDa] | Coverage | Unique peptides | Score |
|---|---|---|---|---|---|
| **E1BFN5** | Uncharacterized protein | 78.333 | 4.803493 | 3 | 4.643848658 |
| **Q1RML9** | Platelet-activating factor acetylhydrolas | 50.119 | 7.882883 | 3 | 8.153270364 |
| **Q05B55** | IGK protein | 26.574 | 12.08333 | 3 | 10.89561057 |
| **Q32KN8** | Tubulin alpha-3 chain | 49.894 | 9.333333 | 3 | 7.207318544 |
| **Q6KDN5** | Complement component 3 (Fragment) | 7.414 | 46.03175 | 3 | 10.34847975 |
| **F1MNV5** | Kininogen-1 | 48.391 | 6.192661 | 3 | 9.142725945 |
| **Q17QL7** | KRT15 protein | 48.969 | 7.284768 | 3 | 7.44270277 |
| **K4JR88** | Alpha-2-macroglobulin variant 22 | 77.345 | 34.87699 | 3 | 86.96624315 |
| **B5B0D4** | Major allergen beta-lactoglobulin | 19.956 | 21.34831 | 3 | 5.753308058 |
| **F1MH40** | Uncharacterized protein | 26.318 | 12.08333 | 3 | 10.89561057 |
| **K4JDR8** | Alpha-2-macroglobulin variant 5 | 45.046 | 9.558824 | 3 | 20.09559512 |
| **A1L595** | Keratin, type I cytoskeletal 17 | 48.682 | 9.297052 | 3 | 16.26480794 |
| **A0A140T8C8** | Kininogen-1 | 68.922 | 4.347826 | 3 | 9.142725945 |
| **P01044** | Kininogen-1 | 68.847 | 4.347826 | 3 | 9.142725945 |
| **G3X6I0** | Uncharacterized protein | 202.852 | 2.190923 | 3 | 10.26765418 |
| **F1N045** | Complement component C7 | 92.929 | 3.677343 | 3 | 6.569371939 |
| **Q2HJ49** | Moesin | 67.933 | 4.506066 | 3 | 4.27545166 |
| **F2Z4C1** | Tubulin alpha chain | 50.104 | 9.312639 | 3 | 7.207318544 |
| **P81644** | Apolipoprotein A-II | 11.195 | 16 | 3 | 6.495168686 |
| **A0A140T867** | Keratin, type I cytoskeletal 17 | 48.712 | 9.297052 | 3 | 16.26480794 |
| **Q32LP2** | Radixin | 68.525 | 4.459691 | 3 | 4.27545166 |
| **F1MMP5** | Inter-alpha-trypsin inhibitor heavy chain H1 | 101.174 | 4.856512 | 3 | 8.193274856 |
| **A5PKC2** | SHBG protein | 43.288 | 12.7182 | 3 | 8.049062967 |
| **P56652** | Inter-alpha-trypsin inhibitor heavy chain H3 | 99.489 | 3.142536 | 3 | 4.84053421 |
| **F6RP72** | Tubulin alpha chain | 49.797 | 9.35412 | 3 | 7.207318544 |
| **Q28017** | Platelet-activating factor acetylhydrolas | 50.101 | 7.882883 | 3 | 8.153270364 |
| **O46415** | Ferritin light chain | 19.975 | 28.57143 | 3 | 6.997107744 |
| **M0QVY0** | Uncharacterized protein | 60.767 | 8.056042 | 3 | 20.52165961 |
| **E1BD83** | Proteasome subunit alpha type | 29.184 | 14.94253 | 3 | 10.5631783 |
| **O02717** | Non-muscle myosin heavy chain (Fragment) | 72.327 | 6.4 | 3 | 7.038524389 |
| **Q27991** | Myosin-10 | 228.958 | 2.176113 | 3 | 4.014223576 |
| **P01966** | Hemoglobin subunit alpha | 15.175 | 23.94366 | 3 | 8.103943825 |
| **G5E604** | Uncharacterized protein | 11.051 | 37.38318 | 3 | 69.19117427 |

| Code access | Description* | MW [kDa] | Coverage | Unique peptides | Score |
|---|---|---|---|---|---|
| **P02754** | Beta-lactoglobulin | 19.87 | 21.34831 | 3 | 5.753308058 |
| **K4JBA2** | Alpha-2-macroglobulin variant 9 | 43.75 | 9.823678 | 3 | 20.09559512 |
| **A4FV94** | KRT6A protein | 60.783 | 8.056042 | 3 | 20.52165961 |
| **B0JYP6** | IGK protein | 26.304 | 12.08333 | 3 | 10.89561057 |
| **K4JBR5** | Alpha-2-macroglobulin variant 1 | 115.118 | 23.01741 | 3 | 92.61506844 |
| **Q0VCM5** | Inter-alpha-trypsin inhibitor heavy chain H1 | 101.173 | 4.856512 | 3 | 8.193274856 |
| **P01045** | Kininogen-2 | 68.666 | 4.361874 | 3 | 9.142725945 |
| **Q2TBX6** | Proteasome subunit beta type-1 | 26.229 | 14.93776 | 3 | 7.121225834 |
| **Q05443** | Lumican | 38.732 | 8.187135 | 3 | 5.71127522 |
| **G3N0S9** | Uncharacterized protein | 22.321 | 6.532663 | 3 | 48.70267165 |
| **P34955** | Alpha-1-antiproteinase | 46.075 | 8.894231 | 3 | 4.713701844 |
| **P19035** | Apolipoprotein C-III | 10.685 | 31.25 | 3 | 8.54306221 |
| **F1MZ96** | Uncharacterized protein | 26.545 | 12.08333 | 3 | 10.89561057 |
| **P33672** | Proteasome subunit beta type-3 | 22.977 | 18.04878 | 3 | 7.05782795 |
| **E1BB91** | Collagen type VI alpha 3 chain | 342.197 | 1.26183 | 3 | 5.710998774 |
| **F2Z4K0** | Tubulin alpha chain | 49.928 | 9.333333 | 3 | 7.207318544 |
| **P63103** | 14-3-3 protein zeta/delta | 27.728 | 18.36735 | 3 | 6.231706738 |
| **F1MJJ8** | Radixin | 68.541 | 4.459691 | 3 | 4.27545166 |
| **G5E589** | Proteasome subunit beta type | 26.301 | 14.93776 | 3 | 7.121225834 |
| **Q3ZCJ7** | Tubulin alpha-1C chain | 49.825 | 9.35412 | 3 | 7.207318544 |
| **Q32LE5** | Isoaspartyl peptidase/L-asparaginase | 32.03 | 11.03896 | 3 | 6.509634972 |
| **K4JDS3** | Alpha-2-macroglobulin variant 10 | 52.304 | 8.245243 | 3 | 20.09559512 |
| **Q687I9** | Purine nucleoside phosphorylase | 32.046 | 10.38062 | 2 | 4.573058486 |
| **F1MVJ8** | Olfactomedin 4 | 57.76 | 6.090373 | 2 | 5.155620337 |
| **F1MJH1** | Gelsolin | 80.653 | 3.283174 | 2 | 2.386954308 |
| **G3X8C8** | Uncharacterized protein | 25.166 | 13.15789 | 2 | 4.573058486 |
| **P62739** | Actin, aortic smooth muscle | 41.982 | 7.161804 | 2 | 7.369176388 |
| **Q28921** | Alpha 1-antichymotrypsin (Fragment) | 28.553 | 7.539683 | 2 | 5.066781759 |
| **Q28007** | Dihydropyrimidine dehydrogenase [NADP(+)] | 111.625 | 2.439024 | 2 | 1.998378515 |
| **Q3SX14** | Gelsolin | 80.681 | 3.283174 | 2 | 2.386954308 |
| **Q9XTA3** | Myocilin | 54.853 | 3.265306 | 2 | 2.534399986 |
| **A2I7M9** | Serpin A3-2 | 46.208 | 4.622871 | 2 | 5.066781759 |
| **B0JYK6** | Alpha-1,4 glucan phosphorylase | 97.227 | 1.781473 | 2 | 5.125619888 |

| Code access | Description* | MW [kDa] | Coverage | Unique peptides | Score |
|---|---|---|---|---|---|
| **Q1JPD0** | Complement component 8, alpha polypeptide (Fragment) | 32.621 | 7.291667 | 2 | 1.965626001 |
| **P80012** | von Willebrand factor (Fragment) | 102.531 | 2.66809 | 2 | 5.914143801 |
| **Q862P9** | Similar to beta actin (Fragment) | 20.968 | 13.82979 | 2 | 4.352330446 |
| **F1MKC4** | Actin, gamma-enteric smooth muscle | 41.898 | 7.180851 | 2 | 7.369176388 |
| **Q2KIH5** | Complement component 8, alpha polypeptide | 66.292 | 3.565365 | 2 | 1.965626001 |
| **G3MXU3** | Collagen type VI alpha 2 chain | 24.991 | 14.41048 | 2 | 6.121668339 |
| **G3N126** | Collagen type VI alpha 3 chain | 134.797 | 2.261712 | 2 | 3.278194427 |
| **A0A140T871** | Glutamate dehydrogenase 1, mitochondrial | 61.592 | 6.238859 | 2 | 4.960487604 |
| **Q58DU5** | Proteasome subunit alpha type-3 | 28.387 | 9.411765 | 2 | 6.971746206 |
| **F1MJ28** | Alpha-1,4 glucan phosphorylase | 97.218 | 1.781473 | 2 | 5.125619888 |
| **Q3T186** | Ribose-5-phosphate isomerase | 28.735 | 11.36364 | 2 | 6.911178589 |
| **Q8SPJ1** | Junction plakoglobin | 81.769 | 3.355705 | 2 | 5.31604743 |
| **A6QQC9** | OLFM4 protein (Fragment) | 49.706 | 7.159353 | 2 | 5.155620337 |
| **Q28908** | Mucin (Fragment) | 54.83 | 8.730159 | 2 | 26.6109134 |
| **F1MX87** | Complement C8 alpha chain | 66.234 | 3.565365 | 2 | 1.965626001 |
| **H9KUV2** | S-methyl-5'-thioadenosine phosphorylase | 32.883 | 10.20408 | 2 | 2.552754164 |
| **G3N1U4** | Serpin A3-3 | 46.127 | 4.622871 | 2 | 5.066781759 |
| **F1MNF8** | Tubulin alpha chain | 49.87 | 6.23608 | 2 | 5.16932559 |
| **A3FJ56** | Kappa casein (Fragment) | 17.836 | 17.5 | 2 | 10.58230782 |
| **Q705V4** | Kappa-casein (Fragment) | 17.693 | 17.61006 | 2 | 10.58230782 |
| **Q0VCX2** | Endoplasmic reticulum chaperone BiP | 72.356 | 3.358779 | 2 | 0 |
| **A5D7L1** | C-type lectin domain containing 11A | 35.593 | 6.17284 | 2 | 4.870718479 |
| **Q3T052** | Inter-alpha-trypsin inhibitor heavy chain H4 | 101.449 | 1.965066 | 2 | 4.293653488 |
| **F1N549** | Dihydropyrimidine dehydrogenas | 111.766 | 2.439024 | 2 | 1.998378515 |
| **G5E534** | Ribose-5-phosphate isomerase | 32.82 | 9.803922 | 2 | 6.911178589 |
| **Q3ZC07** | Actin, alpha cardiac muscle | 41.992 | 7.161804 | 2 | 7.369176388 |
| **Q2KJF1** | Alpha-1B-glycoprotein | 53.52 | 4.970179 | 2 | 4.54270792 |
| **Q5EA67** | Inter-alpha (Globulin) inhibitor H4 (Plasma Kallikrein-sensitive glycoprotein) | 101.446 | 1.965066 | 2 | 4.293653488 |
| **Q3B7M9** | Glycogen phosphorylase | 96.279 | 1.779359 | 2 | 5.125619888 |
| **F1N1I6** | Gelsolin | 85.634 | 3.072983 | 2 | 2.386954308 |
| **O46375** | Transthyretin | 15.717 | 9.52381 | 2 | 7.818257093 |
| **F1N0I3** | Coagulation factor V | 222.078 | 1.167513 | 2 | 5.12988162 |

| Code access | Description* | MW [kDa] | Coverage | Unique peptides | Score |
|---|---|---|---|---|---|
| **P02081** | Hemoglobin fetal subunit beta | 15.849 | 16.55172 | 2 | 20.82257354 |
| **P02668** | Kappa-casein | 21.256 | 14.73684 | 2 | 10.58230782 |
| **B8YB76** | Homogentisat | 49.965 | 6.741573 | 2 | 1.600483537 |
| **Q9TTE1** | Serpin A3-1 | 46.208 | 4.622871 | 2 | 5.066781759 |
| **P81948** | Tubulin alpha-4A chain | 49.892 | 7.142857 | 2 | 4.593021393 |
| **Q3SX06** | Myocilin | 54.854 | 3.265306 | 2 | 2.534399986 |
| **F1MU24** | Alpha-1,4 glucan phosphorylase | 76.125 | 2.248876 | 2 | 5.125619888 |
| **P68138** | Actin, alpha skeletal muscle | 42.024 | 7.161804 | 2 | 7.369176388 |
| **F1MXQ3** | FAM20C, golgi associated secretory pathway kinase | 61.848 | 5.797101 | 2 | 0 |
| **A8YXZ2** | C8G protein | 25.272 | 13.08017 | 2 | 5.495295286 |
| **A2I7N1** | Serpin A3-5 | 46.368 | 4.622871 | 2 | 5.066781759 |
| **Q3ZBS7** | Vitronectin | 53.541 | 5.252101 | 2 | 4.90358305 |
| **D4QBB4** | Globin A1 | 15.944 | 44.13793 | 2 | 32.65024436 |
| **A5D7Q2** | Uncharacterized protein | 51.638 | 3.49076 | 2 | 4.678791761 |
| **M0QVZ6** | Keratin, type II cytoskeletal 5 | 60.629 | 9.598604 | 2 | 28.92585468 |
| **Q2KIH3** | HGD protein (Fragment) | 44.278 | 7.792208 | 2 | 1.600483537 |
| **Q5E9B5** | Actin, gamma-enteric smooth muscle | 41.85 | 7.180851 | 2 | 7.369176388 |
| **Q3ZEJ6** | Serpin A3-3 | 46.297 | 4.622871 | 2 | 5.066781759 |
| **Q58DT9** | Alpha 2 actin | 45.192 | 6.633907 | 2 | 7.369176388 |
| **G3N1Y3** | Uncharacterized protein | 12.966 | 20.51282 | 2 | 20.82257354 |
| **P19858** | L-lactate | 36.574 | 6.626506 | 2 | 4.39744246 |
| **Q9MYV8** | Haptoglobin (Fragment) | 11.232 | 15 | 2 | 3.76282239 |
| **G3N3E4** | Collagen type VI alpha 3 chain | 185.721 | 1.644157 | 2 | 3.278194427 |
| **Q32L76** | Serum amyloid A-4 protein | 14.678 | 20.15504 | 2 | 4.796698689 |
| **G8JKX4** | Actin, aortic smooth muscle | 45.403 | 6.650246 | 2 | 7.369176388 |
| **A0A0M4FJ17** | Kappa-casein (Fragment) | 16.015 | 19.31034 | 2 | 10.58230782 |
| **P55859** | Purine nucleoside phosphorylase | 32.016 | 10.38062 | 2 | 4.573058486 |
| **F1N614** | 78 kDa glucose-regulated protein precursor | 66.224 | 3.666667 | 2 | 0 |
| **A5D7M6** | KRT5 protein | 62.644 | 9.21273 | 2 | 28.92585468 |
| **Q27983** | Alpha1-antichymotrypsin isoform pHHK11 (Fragment) | 22.697 | 9.359606 | 2 | 5.066781759 |
| **A6QPD4** | LOC790886 protein | 45.399 | 6.582278 | 2 | 4.617819786 |
| **F1MMD7** | Inter-alpha-trypsin inhibitor heavy chain H4 | 101.463 | 1.965066 | 2 | 4.293653488 |
| **A4IFM8** | Actin, alpha 1, skeletal muscle | 41.996 | 7.161804 | 2 | 7.369176388 |

| Code access | Description* | MW [kDa] | Coverage | Unique peptides | Score |
|---|---|---|---|---|---|
| **A2I7N0** | Serpin A3-4 | 46.282 | 4.622871 | 2 | 5.066781759 |
| **F1MLF8** | Homogentisat | 49.811 | 6.772009 | 2 | 1.600483537 |
| **P81265** | Polymeric immunoglobulin receptor | 82.383 | 3.698811 | 2 | 5.614470482 |
| **A2I7N2** | Serpin A3-6 | 46.361 | 4.589372 | 2 | 5.066781759 |
| **K4JB97** | Alpha-2-macroglobulin variant 4 | 42.306 | 7.310705 | 2 | 15.2149837 |
| **A0A140T8A9** | Kappa-casein | 21.224 | 14.73684 | 2 | 10.58230782 |
| **Q28107** | Coagulation factor V | 248.828 | 1.040253 | 2 | 5.12988162 |
| **Q0P5J4** | Keratin, type I cytoskeletal 25 | 49.282 | 2.222222 | 2 | 6.499523044 |
| **A5D7J0** | ACTA2 protein | 42.01 | 7.161804 | 2 | 7.369176388 |
| **P00366** | Glutamate dehydrogenas | 61.473 | 6.272401 | 2 | 4.960487604 |
| **Q3ZC00** | Lymphocyte cytosolic protein 1 (L-plastin) | 70.01 | 3.668262 | 2 | 2.719077349 |
| **Q148H6** | Keratin, type I cytoskeletal 28 | 50.744 | 2.155172 | 2 | 6.499523044 |
| **G8JKW7** | Uncharacterized protein | 46.315 | 4.61165 | 2 | 5.066781759 |
| **Q9MZ31** | Fibronectin variable region (Fragment) | 20.365 | 19.35484 | 2 | 5.509114504 |
| **Q2HJB8** | Tubulin alpha-8 chain | 50.022 | 7.126949 | 2 | 4.593021393 |
| **F1MYX5** | Lymphocyte cytosolic protein 1 | 70.068 | 3.668262 | 2 | 2.719077349 |
| **A6QNW3** | PIGR protein | 82.455 | 3.698811 | 2 | 5.614470482 |
| **Q5XQN5** | Keratin, type II cytoskeletal 5 | 62.898 | 9.151414 | 2 | 28.92585468 |
| **P79334** | Glycogen phosphorylase | 97.232 | 1.781473 | 2 | 5.125619888 |
| **Q3MHN2** | Complement component C9 | 61.958 | 4.19708 | 2 | 4.258058071 |

*Classification given by author.
