# Supplementary material for: Extracellular vesicles in infectious diseases caused by protozoan parasites in buffaloes
Source: J Venom Anim Toxins Incl Trop Dis. 2020 May 29;26:e20190067. doi: 10.1590/1678-9199-JVATITD-2019-0067 (PMC7262785; doi:10.1590/1678-9199-JVATITD-2019-0067)
Supplement: Additional file 2. [file 1678-9199-jvatitd-26-e20190067-s2.pdf]

*J Venom Anim Toxins incl Trop Dis*

https://doi.org/10.1590/1678-9199-JVATITD-2019-0067

# Supplementary Material to "Extracellular vesicles in infectious diseases caused by protozoan parasites in buffaloes"

**Additional file 2.** Summary of extracellular vesicle proteins of *Theileria* spp. isolated from serum of control animals present in Fraction F3.

| Code access | Description* | MW [kDa] | Coverage | Unique Peptides | Score |
|---|---|---|---|---|---|
| **E1BNR0** | Apolipoprotein | 515.438 | 23.40705 | 94 | 495.0726119 |
| **Q2UVX4** | Complement C3 | 187.135 | 31.66767 | 52 | 345.5245875 |
| **A0A0F6QNP7** | Complement component 3 | 187.064 | 31.24623 | 51 | 343.8403611 |
| **G3X7A5** | Complement C3 | 187.028 | 30.70439 | 51 | 330.6581188 |
| **B8Y9S9** | Fibronectin 1 | 262.263 | 15.37494 | 24 | 110.1794619 |
| **G5E5A9** | Fibronectin | 271.952 | 14.81033 | 24 | 110.1794619 |
| **P07589** | Fibronectin | 271.983 | 14.81033 | 24 | 110.1794619 |
| **B8Y9T0** | Fibronectin 1 | 248.974 | 16.18165 | 24 | 110.1794619 |
| **P15497** | Apolipoprotein A-I | 30.258 | 47.16981 | 15 | 117.2911120 |
| **Q0VCM4** | Glycogen phosphorylase | 97.394 | 1.527614 | 1 | 0 |
| **F1MI18** | Uncharacterized protein | 165.654 | 19.68876 | 26 | 176.92844569 |
| **F1MJK3** | Uncharacterized protein | 165.506 | 19.66216 | 26 | 176.9284456 |
| **A0A140T897** | Serum albumin | 69.278 | 28.33607 | 16 | 87.0102145 |
| **P02769** | Serum albumin | 69.248 | 28.33607 | 16 | 87.0102145 |
| **P23805** | Conglutinin | 37.971 | 26.68463 | 14 | 380.1768126 |
| **P80457** | Xanthine dehydrogenase/oxidase | 146.696 | 0.750750 | 1 | 2.965109582 |
| **F1MUT3** | Xanthine dehydrogenase/oxidase | 146.669 | 0.750750 | 16 | 2.965109586 |
| **B0JYQ0** | ALB protein | 69.248 | 26.19439 | 15 | 80.409170196 |
| **F1MVP0** | ADAM metallopeptidase with thrombospondin type 1 | 151.374 | 19.67213 | 15 | 120.12379646 |
| **F1N0R5** | von Willebrand factor | 307.475 | 3.596866 | 8 | 26.1033883094 |
| **F1MAV0** | Fibrinogen beta chain | 56.405 | 20.40404 | 8 | 51.431623342 |
| **E1BH06** | Uncharacterized protein | 192.644 | 15.62320 | 10 | 86.6288429 |
| **F5XVA9** | von Willebrand factor | 307.745 | 3.594306 | 8 | 26.10338830 |

| Code access | Description* | MW [kDa] | Coverage | Unique Peptides | Score |
|---|---|---|---|---|---|
| **P02676** | Fibrinogen beta chain | 53.306 | 20.08547 | 7 | 25.59032678 |
| **F1MY85** | Complement C5a anaphylatoxin | 188.927 | 2.444841 | 4 | 8.5798010826 |
| **V6F869** | Apolipoprotein A-I-like | 23.629 | 29.80769 | 7 | 44.303140 |
| **A0A0F6QMJ3** | Complement component 5 | 188.691 | 2.444841 | 4 | 8.5798010 |
| **F1N3Q7** | Apolipoprotein A-IV | 42.963 | 30.52631 | 13 | 71.981267 |
| **Q32PJ2** | Apolipoprotein A-IV | 42.991 | 30.52631 | 13 | 71.981267 |
| **G3N0I4** | Cytosol aminopeptidas | 54.047 | 1.606425 | 1 | 1.9673806 |
| **P00727** | Cytosol aminopeptidas | 56.254 | 1.967380 | 1 | 1.9673802 |
| **Q693V9** | Complement component 3d | 34.422 | 21.45214 | 1 | 87.169953 |
| **Q3MHL4** | Adenosylhomocysteinase 3 | 47.607 | 21.40992 | 7 | 137.36276 |
| **P00432** | Catalase | 59.878 | 2.466793 | 1 | 0 |
| **Q7SIH1** | Alpha-2-macroglobulin | 167.47 | 31.32450 | 10 | 668.51652 |
| **Q95KV5** | Fibronectin | 38.32 | 38.04034 | 9 | 46.092858 |
| **F1MQ37** | Myosin heavy chain 9 | 226.962 | 19.67213 | 13 | 134.312602 |
| **F1MD73** | Uncharacterized protein | 189.978 | 12.50713 | 10 | 35.2686412 |
| **A5PJE3** | Fibrinogen alpha chain | 66.957 | 10.08130 | 6 | 18.8123501 |
| **P02672** | Fibrinogen alpha chain | 66.971 | 10.08130 | 6 | 18.8123501 |
| **A5D9E9** | Complement C1r subcomponent precursor | 80.161 | 5.673758 | 1 | 6.06269646 |
| **Q03247** | Apolipoprotein | 35.958 | 21.51898 | 6 | 50.4440462 |
| **Q2TBU0** | Haptoglobin | 44.831 | 23.19201 | 8 | 48.0335323 |
| **E1BI98** | Collagen type VI alpha 1 chain | 108.603 | 21.867321 | 6 | 124.459278 |
| **G5E513** | Uncharacterized protein | 49.939 | 20 | 6 | 22.3382167 |
| **A6QPX7** | FGB protein (Fragment) | 37.907 | 9.456521 | 5 | 35.100500 |
| **P01030** | Complement C4 | 101.817 | 17.06522 | 9 | 43.73454607 |
| **E1B805** | Uncharacterized protein | 187.149 | 23.192019 | 8 | 48.0335323 |
| **G3X6K8** | Haptoglobin | 44.845 | 25.69327 | 8 | 34.74552003 |
| **A0A140T881** | Apolipoprotein | 36.017 | 21.518987 | 9 | 50.4440462 |
| **G3N3D4** | Potassium channel tetramerization domain containing 12 | 35.701 | 8.5427135 | 3 | 41.012642 |
| **A7YWR0** | Apolipoprotein | 36.051 | 21.51898 | 3 | 21.51898 |
| **E1BFN6** | Dihydropyrimidinase | 56.267 | 4.207119 | 5 | 4.20711974 |
| **P81187** | Complement factor B | 85.312 | 1.7241379 | 1 | 4.834334 |
| **P17697** | Clusterin | 51.081 | 9.1116173 | 3 | 10.1167018 |
| **F1N076** | Ceruloplasmin | 123.731 | 4.792626 | 4 | 12.1857370 |

| Code access | Description* | MW [kDa] | Coverage | Unique Peptides | Score |
|---|---|---|---|---|---|
| **R9QSM8** | Alpha-2-macroglobulin | 133.302 | 32.637729 | 10 | 531.153768 |
| **F1N514** | CD5 antigen-like precursor | 50.305 | 20.75055 | 7 | 16.0244067 |
| **Q3SZZ9** | FGG protein | 49.136 | 18.16091 | 8 | 38.8492791 |
| **Q0VCX1** | Complement C1s subcomponent | 76.56 | 6.386066 | 12 | 12.067846 |
| **F1MJ12** | Complement C1s subcomponent | 77.332 | 6.330935 | 12 | 23.25133181 |
| **F1MGU7** | Fibrinogen gamma-B chain | 50.2 | 17.83295 | 8 | 38.8492790 |
| **Q95KV4** | Fibronectin (Fragment) | 35.464 | 5.184630 | 4 | 5.97265890 |
| **Q3Y5Z3** | Adiponectin | 26.117 | 20.74950 | 8 | 29.8565749 |
| **Q0ZCB4** | Apolipoprotein | 27.057 | 7.9755563 | 3 | 0.98859376 |
| **A2VDY5** | Hydroxysteroid (17-beta) | 28.378 | 8.1481481 | 2 | 4.8307673 |
| **Q9MYP6** | 17-beta-hydroxysteroid dehydrogenas | 28.404 | 24.27707 | 3 | 9.39274930 |
| **A6QNW7** | CD5L protein | 50.179 | 16.837208 | 4 | 8.77830857 |
| **Q0P5J7** | Keratin. type I cuticular Ha5 | 49.905 | 1.538461 | 2 | 2.12892317 |
| **B7FEK7** | 43kDa collectin | 33.594 | 8.411214 | 4 | 129.4997527 |
| **E1BPW6** | Keratin. type I cuticular Ha5 | 49.739 | 1.54867 | 2 | 2.12892317 |
| **Q28178** | Thrombospondin-1 | 129.451 | 10.2564102 | 11 | 66.87595036 |
| **A7E3W2** | Galectin-3-binding protein | 62.087 | 8.6486486 | 5 | 26.75749302 |
| **F1MKG2** | Collagen type VI alpha 2 chain | 109.253 | 2.0447994 | 2 | 5.74746538 |
| **B8Y898** | Malic enzyme | 63.853 | 12.8640776 | 3 | 28.8594936 |
| **P02663** | Alpha-S2-casein | 26.002 | 8.5585585 | 2 | 6.87464993 |
| **F1MC11** | Keratin. type I cytoskeletal 14 | 51.879 | 15.72327 | 6 | 28.12601042 |
| **A6QNZ7** | Keratin 10 | 54.816 | 8.55513 | 4 | 31.8585664 |
| **P12799** | Fibrinogen gamma-B chain | 50.212 | 1.441441 | 7 | 34.8575392 |
| **Q3SYR8** | Immunoglobulin J chain | 17.846 | 22.92993 | 6 | 69.2885639 |
| **F1N3A1** | Thrombospondin-1 | 129.309 | 11.282051 | 1 | 68.986869 |
| **A7E3D5** | Proteasome subunit alpha type (Fragment) | 26.551 | 4.6413502 | 1 | 1.7184752 |
| **K4JR81** | Alpha-2-macroglobulin variant 12 | 61.673 | 21.723518 | 11 | 186.688745 |
| **Q3ZBG0** | Proteasome subunit alpha type-7 | 27.852 | 4.4354838 | 1 | 1.88745493 |
| **F1MSZ6** | Antithrombin-III OS=Bos taurus | 52.407 | 10.96774 | 4 | 15.8486339 |
| **P00735** | Prothrombin | 70.461 | 3.27510 | 1 | 7.94946922 |
| **F1MFY6** | Collectin-43 precursor | 24.905 | 11.02040 | 4 | 129.993746 |
| **Q58D62** | Fetuin-B | 42.636 | 8.485856 | 3 | 41.0936353 |
| **P06394** | Keratin. type I cytoskeletal 10 | 54.815 | 8.555133 | 4 | 31.9936534 |

| Code access | Description* | MW [kDa] | Coverage | Unique Peptides | Score |
|---|---|---|---|---|---|
| **Q3SZJ0** | Argininosuccinate lyase | 52.71 | 10.847453 | 8 | 120.948452 |
| **G5E5C3** | Proteasome subunit alpha type | 27.267 | 10 | 4 | 7.99463420 |
| **F1MM86** | Complement component C6 | 104.455 | 5.955242 | 3 | 0.99374294 |
| **G3N0V2** | Keratin 1 | 63.113 | 9.075907 | 5 | 43.8835422 |
| **O02808** | Von Willebrand factor (Fragment) | 59.57 | 12.99825 | 5 | 130.999273 |
| **Q3SYT3** | Complement C1r subcomponent precursor | 44.528 | 12.992654 | 4 | 15.88864435 |
| **Q2YDE4** | Proteasome subunit alpha type-6 | 27.382 | 20.993752 | 3 | 12.89826425 |
| **F1MTV7** | Argininosuccinate lyase | 52.682 | 2.1515015 | 4 | 238.998765 |
| **B8YB77** | Malic enzyme | 63.332 | 5.9972543 | 5 | 10.993764 |
| **G3X6N3** | Serotransferrin | 77.616 | 7.9864311 | 5 | 9.9864321 |
| **E1BFG1** | Uncharacterized protein | 50.005 | 15.23342 | 2 | 2.12892319 |
| **F1MRZ6** | Tenascin C | 244.461 | 4.9987354 | 3 | 0.987354335 |
| **G5E5T5** | Uncharacterized protein | 42.442 | 4.997353 | 1 | 0.09375343 |
| **Q3SYR5** | Apolipoprotein C-IV | 14.428 | 8.0937635 | 1 | 0.98365343 |
| **Q29RU4** | Complement component C6 | 104.473 | 12.0937354 | 5 | 129.098765 |
| **Q3T063** | Nicotinate-nucleotide pyrophosphorylase | 31.131 | 3.983754 | 4 | 6.09487465 |
| **E1BL29** | Bleomycin hydrolase | 52.896 | 9.98476453 | 4 | 308.948743 |
| **Q29443** | Serotransferrin | 77.703 | 6.9876543 | 5 | 21.947465443 |
| **F1MHB8** | Nicotinate-nucleotide pyrophosphorylase | 31.165 | 10.0948476 | 1 | 0.98765438 |
| **P41361** | Antithrombin-III | 52.314 | 12.094765 | 3 | 11.0946544 |
| **Q71U44** | Fibronectin (Fragment) | 45.802 | 15.96117 | 4 | 16.0987654 |
| **A0JN60** | Tenascin C | 190.961 | 3.180985 | 2 | 1.98476544 |
| **E1BH94** | Peptidoglycan recognition protein 2 | 59.793 | 12.0958765 | 3 | 11.5987654 |
| **F1MRZ5** | Tenascin C | 191.032 | 3.0958765 | 1 | 7.59876543 |
| **Q2KIV9** | Complement C1q subcomponent subunit B | 26.383 | 7.0948765 | 1 | 7.09586532 |
| **Q28194** | Thrombospondin-1 | 25 | 1.98576543 | 2 | 5.09765432 |
| **Q9TUQ0** | Anion exchange protein | 95.582 | 3.987654 | 3 | 11.87643221 |
| **Q148H5** | Keratin. type II cytoskeletal | 57.372 | 1.714285 | 1 | 16.02440679 |
| **Q32KL2** | Proteasome subunit beta type-5 | 28.591 | 15.887654 | 2 | 5.875421187 |
| **P63258** | Actin. cytoplasmic 2 | 41.766 | 10.987653 | 3 | 9.876543215 |
| **B5B3R8** | Alpha S1 casein | 24.427 | 9.9876543 | 3 | 13.87654321 |
| **A0A1B0Z542** | Heat shock 27 kDa protein 1 | 22.231 | 12.987654 | 1 | 0.09876543 |
| **Q9XSW5** | Anion exchange protein | 104.308 | 2.8765432 | 4 | 15.87643221 |

| Code access | Description* | MW [kDa] | Coverage | Unique Peptides | Score |
|---|---|---|---|---|---|
| **F1MNW4** | Inter-alpha-trypsin inhibitor heavy chain H2 | 106.09 | 4.75687103 | 3 | 6.98654432 |
| **Q3MHF7** | S-methyl-5'-thioadenosine phosphorylase | 31.236 | 19.986432 | 3 | 7.9876543 |
| **F1N3V0** | Malic enzyme | 63.746 | 11.986432 | 3 | 12.8765432 |
| **F1MYN5** | Fibulin-1 | 77.434 | 8.087654 | 3 | 8.98765454 |
| **A5D7S8** | Fibulin-1 | 77.478 | 2.987654 | 2 | 3.9876544 |
| **A0A1C9EIX3** | Heat shock protein family B member 1 variant 1 | 22.351 | 3.0987654 | 2 | 8.98765438 |
| **Q58DP7** | Heat shock 27kDa protein 1 | 17.545 | 10.987654 | 3 | 9.98765434 |
| **E9RHW1** | Heat shock 27kDa protein 1 | 22.379 | 9.9876543 | 2 | 7.9876543 |
| **Q3T0X5** | Proteasome subunit alpha type-1 | 29.567 | 43.28358 | 4 | 238.9876543 |
| **P02662** | Alpha-S1-casein | 24.513 | 11.682242 | 2 | 4.09876548 |
| **P60712** | Actin. cytoplasmic 1 | 41.71 | 7.7333333 | 2 | 7.9876924 |
| **P31976** | Ezrin | 68.717 | 6.9876543 | 2 | 4.0898243 |
| **Q3ZCK9** | Proteasome subunit alpha type-4 | 29.465 | 19.9876543 | 4 | 27.9289973 |
| **Q9TUQ1** | Anion exchange protein | 74.3 | 5.987654 | 4 | 9.763430 |
| **A0A1C9EIX6** | Heat shock protein family B member 1 variant 2 | 22.389 | 19.998533 | 3 | 5.9876543 |
| **A5D984** | Pyruvate kinase | 57.912 | 10.998745 | 3 | 120.87654 |
| **Q28085** | Complement factor H | 140.282 | 4.9988543 | 3 | 3.87654365 |
| **F1MC45** | Complement factor H precursor | 96.53 | 9.993425 | 3 | 10.987654 |
| **F1MRD0** | Actin. cytoplasmic 1 | 41.825 | 10.096543 | 2 | 13.9937432 |
| **Q9BGI2** | Peroxiredoxin-4 | 30.722 | 18.1983738 | 4 | 120.09376354 |
| **Q3ZC87** | Pyruvate kinase (Fragment) | 61.389 | 1.0987654 | 2 | 0.987650876 |
| **E1BEL7** | Heat shock protein beta-1 | 22.564 | 2.021975 | 2 | 8.093763543 |
| **Q7M2T6** | Band 3 anion transport protein (Fragments) | 34.3 | 10.983753 | 4 | 186.09837365 |
| **A5D7R6** | ITIH2 protein | 106.12 | 2.0987643 | 2 | 1.987354332 |
| **G3X7S2** | Heat shock protein beta-1 | 17.432 | 4.0876524 | 3 | 4.08754221 |
| **Q1JQB0** | Collagen type VI alpha 2 chain | 97.075 | 2.0976543 | 1 | 0.98765432 |
| **Q6T182** | Sex hormone-binding globulin (Fragment) | 40.069 | 10.087654 | 2 | 7.0987643 |
| **Q29RQ1** | Complement component C7 | 93.029 | 2.9876543 | 3 | 12.9876543 |
| **G5E5H7** | Uncharacterized protein | 19.898 | 23.987654 | 1 | 4.9876543 |
| **K4JF16** | Alpha-2-macroglobulin variant 23 | 101.296 | 5.09871457 | 1 | 19.8632145 |
| **Q2HJ86** | Tubulin alpha-1D chain | 50.251 | 19.9876543 | 1 | 3.986439876 |
| **P81947** | Tubulin alpha-1B chain | 50.12 | 3.9876543 | 1 | 8.09876543 |
| **A3KLR9** | Superoxide dismutase [Cu-Zn] | 26.16 | 12.9876543 | 1 | 10.87654987 |

| Code access | Description* | MW [kDa] | Coverage | Unique Peptides | Score |
|---|---|---|---|---|---|
| **E1BFN5** | Uncharacterized protein | 78.333 | 10.0987654 | 2 | 5.0882533 |
| **Q1RML9** | Platelet-activating factor acetylhydrolas | 50.119 | 12.0937343 | 1 | 0.98764322 |
| **Q05B55** | IGK protein | 26.574 | 10.0987654 | 1 | 11.0987654 |
| **Q32KN8** | Tubulin alpha-3 chain | 49.894 | 9.0987654 | 2 | 6.0947542 |
| **Q6KDN5** | Complement component 3 (Fragment) | 7.414 | 47.094876 | 2 | 0.09464367 |
| **F1MNV5** | Kininogen-1 | 48.391 | 5.984454 | 3 | 28.09476543 |
| **Q17QL7** | KRT15 protein | 48.969 | 12.0987654 | 3 | 110.1794619 |
| **K4JR88** | Alpha-2-macroglobulin variant 22 | 77.345 | 4.0984326 | 1 | 6.2589853 |
| **B5B0D4** | Major allergen beta-lactoglobulin | 19.956 | 2.986421 | 2 | 0.986422467 |
| **F1MH40** | Uncharacterized protein | 26.318 | 2.76542345 | 1 | 1.09876411 |
| **K4JDR8** | Alpha-2-macroglobulin variant 5 | 45.046 | 5.8765431 | 1 | 0.63212345 |
| **A1L595** | Keratin. type I cytoskeletal 17 | 48.682 | 7.7654234 | 1 | 0.95327888 |
| **A0A140T8C8** | Kininogen-1 | 68.922 | 5.9876543 | 1 | 3.9876543 |
| **P01044** | Kininogen-1 | 68.847 | 5.09876 | 1 | 0.14987654 |
| **G3X6I0** | Uncharacterized protein | 202.852 | 1.9876543 | 1 | 0.260093635 |
| **F1N045** | Complement component C7 | 92.929 | 0.677343 | 2 | 12.8753321 |
| **Q2HJ49** | Moesin | 67.933 | 8.0846354 | 1 | 7.05987364 |
| **F2Z4C1** | Tubulin alpha chain | 50.104 | 7.09483765 | 1 | 6.50948736 |
| **P81644** | Apolipoprotein A-II | 11.195 | 2 | 1 | 0.16497433 |
| **A0A140T867** | Keratin. type I cytoskeletal 17 | 48.712 | 3.594306 | 1 | 6.0924317 |
| **Q32LP2** | Radixin | 68.525 | 1.059876 | 1 | 2.0598473 |
| **F1MMP5** | Inter-alpha-trypsin inhibitor heavy chain H1 | 101.174 | 3.098541 | 1 | 2.93753365 |
| **A5PKC2** | SHBG protein | 43.288 | 11.0847354 | 2 | 31.0475233 |
| **P56652** | Inter-alpha-trypsin inhibitor heavy chain H3 | 99.489 | 1.0847354 | 2 | 5.05948736 |
| **F6RP72** | Tubulin alpha chain | 49.797 | 2.054654 | 1 | 2.059847365 |
| **Q28017** | Platelet-activating factor acetylhydrolas | 50.101 | 3.0948376 | 1 | 0.0594873 |
| **O46415** | Ferritin light chain | 19.975 | 32.059487 | 0 | 0.0598476 |
| **M0QVY0** | Uncharacterized protein | 60.767 | 2.0984634 | 1 | 0.02862225 |
| **E1BD83** | Proteasome subunit alpha type | 29.184 | 15.038634 | 1 | 11.34855783 |
| **O02717** | Non-muscle myosin heavy chain (Fragment) | 72.327 | 6.0947432 | 1 | 7.98362433 |
| **Q27991** | Myosin-10 | 228.958 | 0.0598476 | 1 | 4.958735433 |
| **P01966** | Hemoglobin subunit alpha | 15.175 | 3.5167384 | 1 | 8.0594764 |
| **G5E604** | Uncharacterized protein | 11.051 | 7.04752456 | 1 | 69.0584635 |

| Code access | Description* | MW [kDa] | Coverage | Unique Peptides | Score |
|---|---|---|---|---|---|
| **P02754** | Beta-lactoglobulin | 19.87 | 5.4 | 1 | 0.0918625 |
| **K4JBA2** | Alpha-2-macroglobulin variant 9 | 43.75 | 16.59574468 | 1 | 134.4589926 |
| **A4FV94** | KRT6A protein | 60.783 | 2.272727273 | 1 | 2.706366062 |
| **B0JYP6** | IGK protein | 26.304 | 4.166666667 | 1 | 14.54332852 |
| **K4JBR5** | Alpha-2-macroglobulin variant 1 | 115.118 | 2.00729927 | 2 | 1.662858129 |
| **Q0VCM5** | Inter-alpha-trypsin inhibitor heavy chain H1 | 101.173 | 20 | 1 | 4.905816555 |
| **P01045** | Kininogen-2 | 68.666 | 12.93103448 | 1 | 17.31717205 |
| **Q2TBX6** | Proteasome subunit beta type-1 | 26.229 | 2.466793169 | 1 | 0 |
| **Q05443** | Lumican | 38.732 | 17.09401709 | 1 | 138.1611656 |
| **G3N0S9** | Uncharacterized protein | 22.321 | 1.642335766 | 1 | 2.366074562 |
| **P34955** | Alpha-1-antiproteinase | 46.075 | 3.284671533 | 1 | 2.431547165 |
| **P19035** | Apolipoprotein C-III | 10.685 | 1.527614571 | 1 | 0 |
| **F1MZ96** | Uncharacterized protein | 26.545 | 1.62601626 | 1 | 4.03635323 |
| **P33672** | Proteasome subunit beta type-3 | 22.977 | 4.530744337 | 1 | 7.071590424 |
| **E1BB91** | Collagen type VI alpha 3 chain | 342.197 | 1.724137931 | 1 | 4.834334612 |
| **F2Z4K0** | Tubulin alpha chain | 49.928 | 3.643724696 | 1 | 1.762604713 |
| **P63103** | 14-3-3 protein zeta/delta | 27.728 | 8.196721311 | 1 | 3.0535779 |
| **F1MJJ8** | Radixin | 68.541 | 3.542234332 | 1 | 2.346296549 |
| **G5E589** | Proteasome subunit beta type | 26.301 | 4.013377926 | 1 | 3.323835135 |
| **Q3ZCJ7** | Tubulin alpha-1C chain | 49.825 | 1.408450704 | 1 | 2.039117575 |
| **Q32LE5** | Isoaspartyl peptidase/L-asparaginase | 32.03 | 4.013377926 | 1 | 3.323835135 |
| **K4JDS3** | Alpha-2-macroglobulin variant 10 | 52.304 | 1.269035533 | 1 | 2.039117575 |
| **Q687I9** | Purine nucleoside phosphorylase | 32.046 | 4.191616766 | 1 | 2.190802097 |
| **F1MVJ8** | Olfactomedin 4 | 57.76 | 4.191616766 | 1 | 2.190802097 |
| **F1MJH1** | Gelsolin | 80.653 | 3.539823009 | 1 | 0 |
| **G3X8C8** | Uncharacterized protein | 25.166 | 3.921568627 | 1 | 0 |
| **P62739** | Actin. aortic smooth muscle | 41.982 | 0.750750751 | 1 | 2.965109587 |
| **Q28921** | Alpha 1-antichymotrypsin (Fragment) | 28.553 | 0.094746435 | 1 | 3.094763543 |
| **Q28007** | Dihydropyrimidine dehydrogenase [NADP(+)] | 111.625 | 18.8034188 | 1 | 14.98669171 |
| **Q3SX14** | Gelsolin | 80.681 | 15.17241379 | 1 | 14.98669171 |
| **Q9XTA3** | Myocilin | 54.853 | 6.986899563 | 1 | 4.565480947 |
| **A2I7M9** | Serpin A3-2 | 46.208 | 9.142857143 | 1 | 4.565480947 |
| **B0JYK6** | Alpha-1.4 glucan phosphorylase | 97.227 | 5.97826087 | 1 | 2.060161829 |

| Code access | Description* | MW [kDa] | Coverage | Unique Peptides | Score |
|---|---|---|---|---|---|
| **Q1JPD0** | Complement component 8. alpha polypeptide | 32.621 | 5.97826087 | 1 | 2.060161829 |
| **P80012** | von Willebrand factor (Fragment) | 102.531 | 3.375527426 | 1 | 1.675248504 |
| **Q862P9** | Similar to beta actin (Fragment) | 20.968 | 4.854368932 | 1 | 16.78249788 |
| **F1MKC4** | Actin. gamma-enteric smooth muscle | 41.898 | 4.862236629 | 1 | 16.78249788 |
| **Q2KIH5** | Complement component 8. alpha polypeptide | 66.292 | 2.330508475 | 1 | 1.985862494 |
| **G3MXU3** | Collagen type VI alpha 2 chain | 24.991 | 2.330508475 | 1 | 1.985862494 |
| **G3N126** | Collagen type VI alpha 3 chain | 134.797 | 31.25 | 1 | 0 |
| **A0A140T871** | Glutamate dehydrogenase 1. mitochondrial | 61.592 | 6.530612245 | 1 | 0 |
| **Q58DU5** | Proteasome subunit alpha type-3 | 28.387 | 3.703703704 | 1 | 1.745364189 |
| **F1MJ28** | Alpha-1.4 glucan phosphorylase | 97.218 | 3.703703704 | 1 | 1.745364189 |
| **Q3T186** | Ribose-5-phosphate isomerase | 28.735 | 0.948991696 | 1 | 1.911735296 |
| **Q8SPJ1** | Junction plakoglobin | 81.769 | 0.948991696 | 1 | 1.911735296 |
| **A6QQC9** | OLFM4 protein (Fragment) | 49.706 | 1.146788991 | 1 | 0 |
| **Q28908** | Mucin (Fragment) | 54.83 | 1.606425703 | 1 | 1.967380643 |
| **F1MX87** | Complement C8 alpha chain | 66.234 | 1.541425819 | 1 | 1.967380643 |
| **H9KUV2** | S-methyl-5'-thioadenosine phosphorylase | 32.883 | 4.435483871 | 1 | 1.710465074 |
| **G3N1U4** | Serpin A3-3 | 46.127 | 40 | 1 | 1.683246493 |
| **F1MNF8** | Tubulin alpha chain | 49.87 | 4.641350211 | 1 | 1.710465074 |
| **A3FJ56** | Kappa casein (Fragment) | 17.836 | 7.185628743 | 1 | 1.683246493 |
| **Q705V4** | Kappa-casein (Fragment) | 17.693 | 8.108108108 | 1 | 1.683246493 |
| **Q0VCX2** | Endoplasmic reticulum chaperone BiP | 72.356 | 4.214559387 | 1 | 1.710465074 |
| **A5D7L1** | C-type lectin domain containing 11A | 35.593 | 7.441860465 | 1 | 11.34865713 |
| **Q3T052** | Inter-alpha-trypsin inhibitor heavy chain H4 | 101.449 | 7.441860465 | 1 | 11.34865713 |
| **F1N549** | Dihydropyrimidine dehydrogenas | 111.766 | 4.366812227 | 1 | 2.962756634 |
| **G5E534** | Ribose-5-phosphate isomerase | 32.82 | 39.36781609 | 1 | 177.1562679 |
| **Q3ZC07** | Actin. alpha cardiac muscle | 41.992 | 7.00525394 | 1 | 35.08573425 |
| **Q2KJF1** | Alpha-1B-glycoprotein | 53.52 | 7.00525394 | 1 | 35.08573425 |
| **Q5EA67** | Inter-alpha (Globulin) inhibitor H4 (Plasma Kallikrein-sensitive glycoprotein) | 101.446 | 22.5 | 1 | 2.310394764 |
| **Q3B7M9** | Glycogen phosphorylase | 96.279 | 1.181102362 | 1 | 2.475919485 |
| **F1N1I6** | Gelsolin | 85.634 | 10.5 | 1 | 2.576021194 |
| **O46375** | Transthyretin | 15.717 | 15.10791367 | 1 | 28.31982374 |
| **F1N0I3** | Coagulation factor V | 222.078 | 32.69012486 | 1 | 412.5393873 |

| Code access | Description* | MW [kDa] | Coverage | Unique Peptides | Score |
|---|---|---|---|---|---|
| **P02081** | Hemoglobin fetal subunit beta | 15.849 | 3.140096618 | 1 | 2.346296549 |
| **P02668** | Kappa-casein | 21.256 | 3.140096618 | 1 | 2.346296549 |
| **B8YB76** | Homogentisat | 49.965 | 1.449275362 | 1 | 1.972269893 |
| **Q9TTE1** | Serpin A3-1 | 46.208 | 2.064220183 | 1 | 1.972269893 |
| **P81948** | Tubulin alpha-4A chain | 49.892 | 4.010025063 | 1 | 4.602294922 |
| **Q3SX06** | Myocilin | 54.854 | 3.925233645 | 1 | 21.29518712 |
| **F1MU24** | Alpha-1.4 glucan phosphorylase | 76.125 | 5.594405594 | 1 | 2.830938339 |
| **P68138** | Actin. alpha skeletal muscle | 42.024 | 4.929577465 | 1 | 1.777470112 |
| **F1MXQ3** | FAM20C. golgi associated secretory pathway kinase | 61.848 | 4.506437768 | 1 | 24.21742165 |
| **A8YXZ2** | C8G protein | 25.272 | 1.923076923 | 1 | 1.738595843 |
| **A2I7N1** | Serpin A3-5 | 46.368 | 5.66893424 | 1 | 19.4173373 |
| **Q3ZBS7** | Vitronectin | 53.541 | 5.66893424 | 1 | 19.4173373 |
| **D4QBB4** | Globin A1 | 15.944 | 5.353319058 | 1 | 19.4173373 |
| **A5D7Q2** | Uncharacterized protein | 51.638 | 7.00525394 | 1 | 35.08573425 |
| **M0QVZ6** | Keratin. type II cytoskeletal 5 | 60.629 | 12.76595745 | 1 | 6.226527929 |
| **Q2KIH3** | HGD protein (Fragment) | 44.278 | 12.4137931 | 1 | 6.226527929 |
| **Q5E9B5** | Actin. gamma-enteric smooth muscle | 41.85 | 12.67605634 | 1 | 6.226527929 |
| **Q3ZEJ6** | Serpin A3-3 | 46.297 | 13.63636364 | 1 | 6.226527929 |
| **Q58DT9** | Alpha 2 actin | 45.192 | 11.25 | 1 | 6.226527929 |
| **G3N1Y3** | Uncharacterized protein | 12.966 | 9.473684211 | 1 | 6.226527929 |
| **G3MX98** | Keratin 9 | 54.621 | 12.85714286 | 1 | 6.226527929 |
| **Q9MYV8** | Haptoglobin (Fragment) | 11.232 | 11.32075472 | 1 | 6.226527929 |
| **G3N3E4** | Collagen type VI alpha 3 chain | 185.721 | 12.94964029 | 1 | 6.226527929 |
| **Q32L76** | Serum amyloid A-4 protein | 14.678 | 9.473684211 | 1 | 6.226527929 |
| **G8JKX4** | Actin. aortic smooth muscle | 45.403 | 13.23529412 | 1 | 6.226527929 |
| **A0A0M4FJ17** | Kappa-casein (Fragment) | 16.015 | 12.94964029 | 1 | 6.226527929 |
| **P55859** | Purine nucleoside phosphorylase | 32.016 | 1.201602136 | 1 | 2.475919485 |
| **F1N614** | 78 kDa glucose-regulated protein precursor | 66.224 | 2.917771883 | 1 | 5.472857237 |
| **A5D7M6** | KRT5 protein | 62.644 | 2.925531915 | 1 | 5.472857237 |
| **Q27983** | Alpha1-antichymotrypsin isoform pHHK11 (Fragment) | 22.697 | 1.304347826 | 1 | 2.505360842 |
| **A6QPD4** | LOC790886 protein | 45.399 | 1.271186441 | 1 | 2.505360842 |
| **F1MMD7** | Inter-alpha-trypsin inhibitor heavy chain H4 | 101.463 | 2.535211268 | 1 | 2.505360842 |
| **A4IFM8** | Actin. alpha 1. skeletal muscle | 41.996 | 1.271186441 | 1 | 2.505360842 |

| Code access | Description* | MW [kDa] | Coverage | Unique Peptides | Score |
|---|---|---|---|---|---|
| **A2I7N0** | Serpin A3-4 | 46.282 | 5.102040816 | 1 | 0 |
| **F1MLF8** | Homogentisat | 49.811 | 1.199563795 | 1 | 2.096652985 |
| **P81265** | Polymeric immunoglobulin receptor | 82.383 | 8.064516129 | 1 | 6.581345797 |
| **A2I7N2** | Serpin A3-6 | 46.361 | 0.858369099 | 1 | 0 |
| **K4JB97** | Alpha-2-macroglobulin variant 4 | 42.306 | 0.858369099 | 1 | 0 |
| **A0A140T8A9** | Kappa-casein | 21.224 | 1.453957997 | 1 | 1.972269893 |
| **Q28107** | Coagulation factor V | 248.828 | 1.449275362 | 1 | 1.972269893 |
| **Q0P5J4** | Keratin. type I cytoskeletal 25 | 49.282 | 5.617977528 | 1 | 26.10827029 |
| **A5D7J0** | ACTA2 protein | 42.01 | 4.929577465 | 1 | 1.777470112 |
| **P00366** | Glutamate dehydrogenas | 61.473 | 1.717305152 | 1 | 2.41721487 |
| **Q3ZC00** | Lymphocyte cytosolic protein 1 (L-plastin) | 70.01 | 2.41187384 | 1 | 2.41721487 |
| **Q148H6** | Keratin. type I cytoskeletal 28 | 50.744 | 4.87012987 | 1 | 29.03050482 |
| **G8JKW7** | Uncharacterized protein | 46.315 | 2.917771883 | 1 | 5.472857237 |
| **Q9MZ31** | Fibronectin variable region (Fragment) | 20.365 | 1.923076923 | 1 | 1.738595843 |
| **Q2HJB8** | Tubulin alpha-8 chain | 50.022 | 1.19047619 | 1 | 2.475919485 |
| **F1MYX5** | Lymphocyte cytosolic protein 1 | 70.068 | 3.867403315 | 1 | 24.21742165 |
| **A6QNW3** | PIGR protein | 82.455 | 39.408867 | 1 | 187.7336295 |
| **Q5XQN5** | Keratin. type II cytoskeletal 5 | 62.898 | 2.709359606 | 1 | 5.472857237 |
| **P79334** | Glycogen phosphorylase | 97.232 | 2.925531915 | 1 | 5.472857237 |
| **Q3MHN2** | Complement component C9 | 61.958 | 2.917771883 | 1 | 5.472857237 |

*Classification given by author.
