# Supplementary material for: Extracellular vesicles in infectious diseases caused by protozoan parasites in buffaloes
Source: J Venom Anim Toxins Incl Trop Dis. 2020 May 29;26:e20190067. doi: 10.1590/1678-9199-JVATITD-2019-0067 (PMC7262785; doi:10.1590/1678-9199-JVATITD-2019-0067)
Supplement: Additional file 3. [file 1678-9199-jvatitd-26-e20190067-s3.pdf]

J Venom Anim Toxins incl Trop Dis

https://doi.org/10.1590/1678-9199-JVATITD-2019-0067

Supplementary Material to "Extracellular vesicles in infectious diseases caused by protozoan parasites in buffaloes"

Additional file 3. Summary of extracellular vesicle proteins of *Theileria* spp. isolated from serum of treated animals present in Fraction F3.

Code access	Description*	MW [kDa]	Coverage	Unique peptides	Score
**E1BNR0**	Apolipoprotein	515.438	24.12962557	103	674,8379145
**Q2UVX4**	Complement C3	187.135	33.65442505	56	440,4326642
**A0A0F6QNP7**	Complement component 3	187.064	33.23299217	55	440,4326642
**G3X7A5**	Complement C3	187.028	32.69114991	55	410,7775776
**B8Y9S9**	Fibronectin 1	262.263	27.06325932	3	327,4399574
**G5E5A9**	Fibronectin	271.952	25.14124294	2	321,4439662
**P07589**	Fibronectin	271.983	25.14124294	42	321,4439662
**B8Y9T0**	Fibronectin 1	248.974	26.54320988	21	318,3170657
**P15497**	Apolipoprotein A-I	30.258	19.7564276	24	220,1763519
**Q0VCM4**	Glycogen phosphorylase	97.394	19.72972973	38	220,1763519
**F1MI18**	Uncharacterized protein	165.654	59.62264151	22	232,1050236
**F1MJK3**	Uncharacterized protein	165.506	8.790035587	0	115,8492713
**A0A140T897**	Serum albumin	69.278	8.796296296	19	115,8492713
**P02769**	Serum albumin	69.248	27.22371968	5	404,0782466
**P23805**	Conglutinin	37.971	26.1589404	3	873,9068512
**P80457**	Xanthine dehydrogenase/oxidase	146.696	14,44444444	14	65,70898986
**F1MUT3**	Xanthine dehydrogenase/oxidase	146.669	22,73476112	9	73,49145103
**B0JYQ0**	ALB protein	69.248	22,73476112	15	73,49145103
**F1MVP0**	ADAM metallopeptidase with thrombospondin type 1	151.374	48,55769231	13	119,6688337
**F1N0R5**	von Willebrand factor	307.475	15,57377049	0	100,6843615
**F1MAV0**	Fibrinogen beta chain	56.405	13,41880342	12	62,5247829
**E1BH06**	Uncharacterized protein	192.644	30,3030303	10	52,56129336
**F5XVA9**	von Willebrand factor	307.745	32,05128205	5	52,56129336

Code access	Description*	MW [kDa]	Coverage	Unique peptides	Score
**P02676**	Fibrinogen beta chain	53.306	20,59308072	6	62,91113496
**F1MY85**	Complement C5a anaphylatoxin	188.927	13,60655738	3	100,6843615
**V6F869**	Apolipoprotein A-I-like	23.629	44,6685879	12	108,6491276
**A0A0F6QMJ3**	Complement component 5	188.691	25,67324955	8	303,3408718
**F1N3Q7**	Apolipoprotein A-IV	42.963	8,737864078	0	71,69110668
**Q32PJ2**	Apolipoprotein A-IV	42.991	19,0070922	15	31,68743467
**G3N0I4**	Cytosol aminopeptidas	54.047	13,95749569	10	111,2448874
**P00727**	Cytosol aminopeptidas	56.254	26,31578947	3	33,63287568
**Q693V9**	Complement component 3d	34.422	26,31578947	10	33,63287568
**Q3MHL4**	Adenosylhomocysteinase 3	47.607	30,0330033	2	76,51224542
**P00432**	Catalase	59.878	12,44239631	9	25,84321988
**Q7SIH1**	Alpha-2-macroglobulin	167.47	24,29048414	3	646,0610726
**Q95KV5**	Fibronectin	38.32	6,357388316	5	20,59954548
**F1MQ37**	Myosin heavy chain 9	226.962	6,357388316	4	20,59954548
**F1MD73**	Uncharacterized protein	189.978	4,975347378	8	20,59954548
**A5PJE3**	Fibrinogen alpha chain	66.957	38,75	0	71,74341667
**P02672**	Fibrinogen alpha chain	66.971	14,77477477	5	62,25517595
**A5D9E9**	Complement C1r subcomponent precursor	80.161	13,49593496	9	39,98661864
**Q03247**	Apolipoprotein	35.958	13,49593496	8	39,98661864
**Q2TBU0**	Haptoglobin	44.831	27,84810127	0	25,54059851
**E1BI98**	Collagen type VI alpha 1 chain	108.603	27,84810127	4	25,54059851
**G5E513**	Uncharacterized protein	49.939	27,84810127	6	25,54059851
**A6QPX7**	FGB protein (Fragment)	37.907	28,48484848	8	30,96007919
**P01030**	Complement C4	101.817	28,18627451	9	267,9672889
**E1B805**	Uncharacterized protein	187.149	28,96725441	12	267,9672889
**G3X6K8**	Haptoglobin	44.845	24,31289641	15	267,9672889
**A0A140T881**	Apolipoprotein	36.017	10,21582734	7	32,74262905
**G3N3D4**	Potassium channel tetramerization domain containing	35.701	10,30478955	7	32,74262905
**A7YWR0**	Apolipoprotein	36.051	14,4469526	19	38,56606507
**E1BFN6**	Dihydropyrimidinase	56.267	14,71264368	20	38,56606507
**P81187**	Complement factor B	85.312	27,154047	0	227,8457786
**P17697**	Clusterin	51.081	17,30418944	13	42,44953275
**F1N076**	Ceruloplasmin	123.731	36,24454148	7	32,37882257

Code access	Description*	MW [kDa]	Coverage	Unique peptides	Score
**R9QSM8**	Alpha-2-macroglobulin	133.302	25,43520309	2	505,1839079
**F1N514**	CD5 antigen-like precursor	50.305	32,38512035	0	770,8895055
**Q3SZZ9**	FGG protein	49.136	22,92993631	12	60,16956151
**Q0VCX1**	Complement C1s subcomponent	76.56	5,501618123	9	11,86303473
**F1MJ12**	Complement C1s subcomponent	77.332	13,30798479	3	32,08415091
**F1MGU7**	Fibrinogen gamma-B chain	50.2	13,30798479	5	32,08415091
**Q95KV4**	Fibronectin (Fragment)	35.464	31,64556962	1	14,37422311
**Q3Y5Z3**	Adiponectin	26.117	11,03603604	0	33,17564273
**Q0ZCB4**	Apolipoprotein	27.057	27,27272727	3	148,6272044
**A2VDY5**	Hydroxysteroid (17-beta)	28.378	20,38834951	9	65,30266976
**Q9MYP6**	17-beta-hydroxysteroid dehydrogenas	28.404	25,41666667	19	22,7700392
**A6QNW7**	CD5L protein	50.179	10,93394077	5	23,2725662
**Q0P5J7**	Keratin. type I cuticular Ha5	49.905	12,14128035	21	87,20624363
**B7FEK7**	43kDa collectin	33.594	12,19512195	18	87,20624363
**E1BPW6**	Keratin. type I cuticular Ha5	49.739	10,54347826	7	51,87173843
**Q28178**	Thrombospondin-1	129.451	28,76106195	5	551,6399485
**A7E3W2**	Galectin-3-binding protein	62.087	8,333333333	10	14,14523625
**F1MKG2**	Collagen type VI alpha 2 chain	109.253	17,81376518	3	26,02097392
**B8Y898**	Malic enzyme	63.853	12,40875912	6	23,08411241
**P02663**	Alpha-S2-casein	26.002	7,755775578	7	24,12152457
**F1MC11**	Keratin. type I cytoskeletal 14	51.879	48,59813084	9	65,37755024
**A6QNZ7**	Keratin 10	54.816	5,176767677	10	9,596704483
**P12799**	Fibrinogen gamma-B chain	50.212	12,43654822	23	11,27226317
**Q3SYR8**	Immunoglobulin J chain	17.846	9,009009009	0	5,487135172
**F1N3A1**	Thrombospondin-1	129.309	9,009009009	1	5,487135172
**A7E3D5**	Proteasome subunit alpha type (Fragment)	26.551	4,856512141	0	10,87347269
**K4JR81**	Alpha-2-macroglobulin variant 12	61.673	4,856512141	3	10,87347269
**Q3ZBG0**	Proteasome subunit alpha type-7	27.852	8,411214953	2	126,5844053
**F1MSZ6**	Antithrombin-III OS=Bos taurus	52.407	11,02040816	9	126,5844053
**P00735**	Prothrombin	70.461	36,24733475	4	260,5843923
**F1MFY6**	Collectin-43 precursor	24.905	34,70437018	3	846,0876623
**Q58D62**	Fetuin-B	42.636	3,310613437	0	7,906161785
**P06394**	Keratin. type I cytoskeletal 10	54.815	6,204379562	2	6,61307168

Code access	Description*	MW [kDa]	Coverage	Unique peptides	Score
**Q3SZJ0**	Argininosuccinate lyase	52.71	4,744525547	0	6,086005092
**G5E5C3**	Proteasome subunit alpha type	27.267	10,57692308	1	4,239753962
**F1MM86**	Complement component C6	104.455	2,702702703	7	3,410375118
**G3N0V2**	Keratin 1	63.113	11,07954545	0	4,860876799
**O02808**	Von Willebrand factor (Fragment)	59.57	11,07954545	9	4,860876799
**Q3SYT3**	Complement C1r subcomponent precursor	44.528	5,113636364	10	7,11321342
**Q2YDE4**	Proteasome subunit alpha type-6	27.382	11,11111111	0	6,825133801
**F1MTV7**	Argininosuccinate lyase	52.682	11,11111111	1	6,825133801
**B8YB77**	Malic enzyme	63.332	5,812417437	4	7,035996199
**G3X6N3**	Serotransferrin	77.616	5,812417437	3	7,035996199
**E1BFG1**	Uncharacterized protein	50.005	4,756871036	0	11,78225541
**F1MRZ6**	Tenascin C	244.461	4,756871036	4	11,78225541
**G5E5T5**	Uncharacterized protein	42.442	5,113636364	1	7,11321342
**Q3SYR5**	Apolipoprotein C-IV	14.428	5,38573508	2	4,48806572
**Q29RU4**	Complement component C6	104.473	23,07692308	1	11,25674486
**Q3T063**	Nicotinate-nucleotide pyrophosphorylase	31.131	4,347826087	2	4,48806572
**E1BL29**	Bleomycin hydrolase	52.896	6,532663317	0	27,89325523
**Q29443**	Serotransferrin	77.703	20,15503876	2	3,950842738
**F1MHB8**	Nicotinate-nucleotide pyrophosphorylase	31.165	8,558558559	2	1,680921078
**P41361**	Antithrombin-III	52.314	12,5	1	110,3163168
**Q71U44**	Fibronectin (Fragment)	45.802	17,59656652	1	109,0141351
**A0JN60**	Tenascin C	190.961	20,47244094	2	5,039208889
**E1BH94**	Peptidoglycan recognition protein 2	59.793	23,95833333	0	7,306949139
**F1MRZ5**	Tenascin C	191.032	6,28742515	2	4,237655163
**Q2KIV9**	Complement C1q subcomponent subunit B	26.383	6,290672451	0	7,969627857
**Q28194**	Thrombospondin-1	25	1,493428913	2	4,015279174
**Q9TUQ0**	Anion exchange protein	95.582	6,982543641	10	2,994791746
**Q148H5**	Keratin. type II cytoskeletal	57.372	7,00525394	2	16,71615529
**Q32KL2**	Proteasome subunit beta type-5	28.591	7,00525394	6	16,71615529
**P63258**	Actin. cytoplasmic 2	41.766	4,22832981	2	4,367512226
**B5B3R8**	Alpha S1 casein	24.427	4,22832981	0	4,367512226
**A0A1B0Z542**	Heat shock 27 kDa protein 1	22.231	1,431127013	3	4,702090144
**Q9XSW5**	Anion exchange protein	104.308	5,735660848	0	9,489186764

Code access	Description*	MW [kDa]	Coverage	Unique peptides	Score
**F1MNW4**	Inter-alpha-trypsin inhibitor heavy chain H2	106.09	5,735660848	3	9,489186764
**Q3MHF7**	S-methyl-5'-thioadenosine phosphorylase	31.236	3,565062389	2	4,589993954
**F1N3V0**	Malic enzyme	63.746	3,584229391	1	4,589993954
**F1MYN5**	Fibulin-1	77.434	2,253855279	0	4,057821274
**A5D7S8**	Fibulin-1	77.478	2,253855279	3	4,057821274
**A0A1C9EIX3**	Heat shock protein family B member 1 variant 1	22.351	2,549575071	12	4,26041913
**Q58DP7**	Heat shock 27kDa protein 1	17.545	2,549575071	0	4,26041913
**E9RHW1**	Heat shock 27kDa protein 1	22.379	18,46153846	1	6,605324507
**Q3T0X5**	Proteasome subunit alpha type-1	29.567	18,46153846	0	6,605324507
**P02662**	Alpha-S1-casein	24.513	0,788643533	0	7,681156635
**P60712**	Actin. cytoplasmic 1	41.71	1,947419669	2	7,787001133
**P31976**	Ezrin	68.717	7,733333333	1	7,858686447
**Q3ZCK9**	Proteasome subunit alpha type-4	29.465	7,733333333	0	7,858686447
**Q9TUQ1**	Anion exchange protein	74.3	7,733333333	5	7,858686447
**A0A1C9EIX6**	Heat shock protein family B member 1 variant 2	22.389	1,431127013	3	4,702090144
**A5D984**	Pyruvate kinase	57.912	15,10791367	1	11,06255352
**Q28085**	Complement factor H	140.282	12,93103448	2	12,63318157
**F1MC45**	Complement factor H precursor	96.53	13,67521364	3	12,63318157
**F1MRD0**	Actin. cytoplasmic 1	41.825	10	2	12,63318157
**Q9BGI2**	Peroxiredoxin-4	30.722	12,76595744	0	12,63318157
**Q3ZC87**	Pyruvate kinase (Fragment)	61.389	27,06222865	1	453,5852154
**E1BEL7**	Heat shock protein beta-1	22.564	32,60869565	1	5,436547518
**Q7M2T6**	Band 3 anion transport protein (Fragments)	34.3	32,78688525	2	5,436547518
**A5D7R6**	ITIH2 protein	106.12	19,53125	1	5,436547518
**G3X7S2**	Heat shock protein beta-1	17.432	32,78688525	1	5,436547518
**Q1JQB0**	Collagen type VI alpha 2 chain	97.075	9,693877551	1	5,790735483
**Q6T182**	Sex hormone-binding globulin (Fragment)	40.069	6,181015453	0	6,662236333
**Q29RQ1**	Complement component C7	93.029	19,35483871	2	12,07949877
**G5E5H7**	Uncharacterized protein	19.898	16,02564103	0	5,436547518
**K4JF16**	Alpha-2-macroglobulin variant 23	101.296	16,25615764	1	21,39780414
**Q2HJ86**	Tubulin alpha-1D chain	50.251	3,434610304	3	4,737163544
**P81947**	Tubulin alpha-1B chain	50.12	2,599814299	5	2,994791746
**A3KLR9**	Superoxide dismutase [Cu-Zn]	26.16	2,094240838	0	2,994791746

Code access	Description*	MW [kDa]	Coverage	Unique peptides	Score
**E1BFN5**	Uncharacterized protein	78.333	24,12962557	1	674,8379145
**Q1RML9**	Platelet-activating factor acetylhydrolas	50.119	33,65442505	1	440,4326642
**Q05B55**	IGK protein	26.574	33,23299217	1	440,4326642
**Q32KN8**	Tubulin alpha-3 chain	49.894	32,69114991	2	410,7775776
**Q6KDN5**	Complement component 3 (Fragment)	7.414	27,06325932	2	327,4399574
**F1MNV5**	Kininogen-1	48.391	25,14124294	0	321,4439662
**Q17QL7**	KRT15 protein	48.969	25,14124294	0	321,4439662
**K4JR88**	Alpha-2-macroglobulin variant 22	77.345	26,54320988	2	318,3170657
**B5B0D4**	Major allergen beta-lactoglobulin	19.956	19,7564276	2	220,1763519
**F1MH40**	Uncharacterized protein	26.318	19,72972973	1	220,1763519
**K4JDR8**	Alpha-2-macroglobulin variant 5	45.046	59,62264151	2	232,1050236
**A1L595**	Keratin. type I cytoskeletal 17	48.682	8,790035587	2	115,8492713
**A0A140T8C8**	Kininogen-1	68.922	8,796296296	2	115,8492713
**P01044**	Kininogen-1	68.847	27,22371968	0	404,0782466
**G3X6I0**	Uncharacterized protein	202.852	26,1589404	3	873,9068512
**F1N045**	Complement component C7	92.929	14,44444444	2	65,70898986
**Q2HJ49**	Moesin	67.933	22,73476112	0	73,49145103
**F2Z4C1**	Tubulin alpha chain	50.104	22,73476112	2	73,49145103
**P81644**	Apolipoprotein A-II	11.195	48,55769231	3	119,6688337
**A0A140T867**	Keratin. type I cytoskeletal 17	48.712	15,57377049	2	100,6843615
**Q32LP2**	Radixin	68.525	13,41880342	3	62,5247829
**F1MMP5**	Inter-alpha-trypsin inhibitor heavy chain H1	101.174	30,3030303	0	52,56129336
**A5PKC2**	SHBG protein	43.288	32,05128205	2	52,56129336
**P56652**	Inter-alpha-trypsin inhibitor heavy chain H3	99.489	20,59308072	1	62,91113496
**F6RP72**	Tubulin alpha chain	49.797	13,60655738	2	100,6843615
**Q28017**	Platelet-activating factor acetylhydrolas	50.101	44,6685879	1	108,6491276
**O46415**	Ferritin light chain	19.975	25,67324955	0	303,3408718
**M0QVY0**	Uncharacterized protein	60.767	8,737864078	1	71,69110668
**E1BD83**	Proteasome subunit alpha type	29.184	19,0070922	1	31,68743467
**O02717**	Non-muscle myosin heavy chain (Fragment)	72.327	13,95749569	1	111,2448874
**Q27991**	Myosin-10	228.958	26,31578947	1	33,63287568
**P01966**	Hemoglobin subunit alpha	15.175	26,31578947	1	33,63287568
**G5E604**	Uncharacterized protein	11.051	30,0330033	3	76,51224542

Code access	Description*	MW [kDa]	Coverage	Unique peptides	Score
**P02754**	Beta-lactoglobulin	19.87	12,44239631	1	25,84321988
**K4JBA2**	Alpha-2-macroglobulin variant 9	43.75	24,29048414	2	646,0610726
**A4FV94**	KRT6A protein	60.783	6,357388316	0	20,59954548
**B0JYP6**	IGK protein	26.304	6,357388316	1	20,59954548
**K4JBR5**	Alpha-2-macroglobulin variant 1	115.118	4,975347378	0	20,59954548
**Q0VCM5**	Inter-alpha-trypsin inhibitor heavy chain H1	101.173	38,75	0	71,74341667
**P01045**	Kininogen-2	68.666	14,77477477	1	62,25517595
**Q2TBX6**	Proteasome subunit beta type-1	26.229	13,49593496	0	39,98661864
**Q05443**	Lumican	38.732	13,49593496	2	39,98661864
**G3N0S9**	Uncharacterized protein	22.321	27,84810127	1	25,54059851
**P34955**	Alpha-1-antiproteinase	46.075	27,84810127	1	25,54059851
**P19035**	Apolipoprotein C-III	10.685	27,84810127	1	25,54059851
**F1MZ96**	Uncharacterized protein	26.545	28,48484848	1	30,96007919
**P33672**	Proteasome subunit beta type-3	22.977	28,18627451	2	267,9672889
**E1BB91**	Collagen type VI alpha 3 chain	342.197	28,96725441	1	267,9672889
**F2Z4K0**	Tubulin alpha chain	49.928	24,31289641	1	267,9672889
**P63103**	14-3-3 protein zeta/delta	27.728	10,21582734	1	32,74262905
**F1MJJ8**	Radixin	68.541	10,30478955	4	32,74262905
**G5E589**	Proteasome subunit beta type	26.301	14,4469526	0	38,56606507
**Q3ZCJ7**	Tubulin alpha-1C chain	49.825	14,71264368	1	38,56606507
**Q32LE5**	Isoaspartyl peptidase/L-asparaginase	32.03	27,154047	2	227,8457786
**K4JDS3**	Alpha-2-macroglobulin variant 10	52.304	17,30418944	2	42,44953275
**Q687I9**	Purine nucleoside phosphorylase	32.046	36,24454148	1	32,37882257
**F1MVJ8**	Olfactomedin 4	57.76	25,43520309	0	505,1839079
**F1MJH1**	Gelsolin	80.653	32,38512035	1	770,8895055
**G3X8C8**	Uncharacterized protein	25.166	22,92993631	0	60,16956151
**P62739**	Actin. aortic smooth muscle	41.982	5,501618123	4	11,86303473
**Q28921**	Alpha 1-antichymotrypsin (Fragment)	28.553	13,30798479	3	32,08415091
**Q28007**	Dihydropyrimidine dehydrogenase [NADP(+)]	111.625	13,30798479	2	32,08415091
**Q3SX14**	Gelsolin	80.681	31,64556962	1	14,37422311
**Q9XTA3**	Myocilin	54.853	11,03603604	1	33,17564273
**A2I7M9**	Serpin A3-2	46.208	27,27272727	1	148,6272044
**B0JYK6**	Alpha-1.4 glucan phosphorylase	97.227	20,38834951	2	65,30266976

Code access	Description*	MW [kDa]	Coverage	Unique peptides	Score
**Q1JPD0**	Complement component 8. alpha polypeptide	32.621	25,41666667	1	22,7700392
**P80012**	von Willebrand factor (Fragment)	102.531	10,93394077	1	23,2725662
**Q862P9**	Similar to beta actin (Fragment)	20.968	12,14128035	1	87,20624363
**F1MKC4**	Actin. gamma-enteric smooth muscle	41.898	12,19512195	0	87,20624363
**Q2KIH5**	Complement component 8. alpha polypeptide	66.292	10,54347826	3	51,87173843
**G3MXU3**	Collagen type VI alpha 2 chain	24.991	28,76106195	2	551,6399485
**G3N126**	Collagen type VI alpha 3 chain	134.797	8,333333333	0	14,14523625
**A0A140T871**	Glutamate dehydrogenase 1. mitochondrial	61.592	17,81376518	1	26,02097392
**Q58DU5**	Proteasome subunit alpha type-3	28.387	12,40875912	4	23,08411241
**F1MJ28**	Alpha-1.4 glucan phosphorylase	97.218	7,755775578	6	24,12152457
**Q3T186**	Ribose-5-phosphate isomerase	28.735	48,59813084	3	65,37755024
**Q8SPJ1**	Junction plakoglobin	81.769	5,176767677	0	9,596704483
**A6QQC9**	OLFM4 protein (Fragment)	49.706	12,43654822	2	11,27226317
**Q28908**	Mucin (Fragment)	54.83	9,009009009	2	5,487135172
**F1MX87**	Complement C8 alpha chain	66.234	9,009009009	1	5,487135172
**H9KUV2**	S-methyl-5'-thioadenosine phosphorylase	32.883	4,856512141	0	10,87347269
**G3N1U4**	Serpin A3-3	46.127	4,856512141	1	10,87347269
**F1MNF8**	Tubulin alpha chain	49.87	8,411214953	1	126,5844053
**A3FJ56**	Kappa casein (Fragment)	17.836	11,02040816	1	126,5844053
**Q705V4**	Kappa-casein (Fragment)	17.693	36,24733475	0	260,5843923
**Q0VCX2**	Endoplasmic reticulum chaperone BiP	72.356	34,70437018	3	846,0876623
**A5D7L1**	C-type lectin domain containing 11A	35.593	3,310613437	0	7,906161785
**Q3T052**	Inter-alpha-trypsin inhibitor heavy chain H4	101.449	6,204379562	2	6,61307168
**F1N549**	Dihydropyrimidine dehydrogenas	111.766	4,744525547	4	6,086005092
**G5E534**	Ribose-5-phosphate isomerase	32.82	10,57692308	3	4,239753962
**Q3ZC07**	Actin. alpha cardiac muscle	41.992	2,702702703	2	3,410375118
**Q2KJF1**	Alpha-1B-glycoprotein	53.52	11,07954545	3	4,860876799
**Q5EA67**	Inter-alpha (Globulin) inhibitor H4 (Plasma Kallikrein-sensitive glycoprotein)	101.446	11,07954545	2	4,860876799
**Q3B7M9**	Glycogen phosphorylase	96.279	5,113636364	1	7,11321342
**F1N1I6**	Gelsolin	85.634	11,11111111	3	6,825133801
**O46375**	Transthyretin	15.717	11,11111111	5	6,825133801
**F1N0I3**	Coagulation factor V	222.078	5,812417437	3	7,035996199

Code access	Description*	MW [kDa]	Coverage	Unique peptides	Score
**P02081**	Hemoglobin fetal subunit beta	15.849	5,812417437	2	7,035996199
**P02668**	Kappa-casein	21.256	4,756871036	3	11,78225541
**B8YB76**	Homogentisat	49.965	4,756871036	2	11,78225541
**Q9TTE1**	Serpin A3-1	46.208	5,113636364	3	7,11321342
**P81948**	Tubulin alpha-4A chain	49.892	5,38573508	2	4,48806572
**Q3SX06**	Myocilin	54.854	23,07692308	3	11,25674486
**F1MU24**	Alpha-1.4 glucan phosphorylase	76.125	4,347826087	3	4,48806572
**P68138**	Actin. alpha skeletal muscle	42.024	6,532663317	2	27,89325523
**F1MXQ3**	FAM20C. golgi associated secretory pathway kinase	61.848	20,15503876	1	3,950842738
**A8YXZ2**	C8G protein	25.272	8,558558559	2	1,680921078
**A2I7N1**	Serpin A3-5	46.368	12,5	0	110,3163168
**Q3ZBS7**	Vitronectin	53.541	17,59656652	1	109,0141351
**D4QBB4**	Globin A1	15.944	20,47244094	1	5,039208889
**A5D7Q2**	Uncharacterized protein	51.638	23,95833333	1	7,306949139
**M0QVZ6**	Keratin. type II cytoskeletal 5	60.629	6,28742515	1	4,237655163
**Q2KIH3**	HGD protein (Fragment)	44.278	6,290672451	1	7,969627857
**Q5E9B5**	Actin. gamma-enteric smooth muscle	41.85	1,493428913	1	4,015279174
**Q3ZEJ6**	Serpin A3-3	46.297	6,982543641	1	2,994791746
**Q58DT9**	Alpha 2 actin	45.192	7,00525394	1	16,71615529
**G3N1Y3**	Uncharacterized protein	12.966	7,00525394	1	16,71615529
**G3MX98**	Keratin 9	54.621	4,22832981	1	4,367512226
**Q9MYV8**	Haptoglobin (Fragment)	11.232	4,22832981	1	4,367512226
**G3N3E4**	Collagen type VI alpha 3 chain	185.721	1,431127013	1	4,702090144
**Q32L76**	Serum amyloid A-4 protein	14.678	5,735660848	1	9,489186764
**G8JKX4**	Actin. aortic smooth muscle	45.403	5,735660848	1	9,489186764
**A0A0M4FJ17**	Kappa-casein (Fragment)	16.015	3,565062389	1	4,589993954
**P55859**	Purine nucleoside phosphorylase	32.016	3,584229391	1	4,589993954
**F1N614**	78 kDa glucose-regulated protein precursor	66.224	2,253855279	1	4,057821274
**A5D7M6**	KRT5 protein	62.644	2,253855279	1	4,057821274
**Q27983**	Alpha1-antichymotrypsin isoform pHHK11 (Fragment)	22.697	2,549575071	1	4,26041913
**A6QPD4**	LOC790886 protein	45.399	2,549575071	1	4,26041913
**F1MMD7**	Inter-alpha-trypsin inhibitor heavy chain H4	101.463	18,46153846	1	6,605324507
**A4IFM8**	Actin. alpha 1. skeletal muscle	41.996	18,46153846	1	6,605324507

Code access	Description*	MW [kDa]	Coverage	Unique peptides	Score
**A2I7N0**	Serpin A3-4	46.282	0,788643533	1	7,681156635
**F1MLF8**	Homogentisat	49.811	1,947419669	1	7,787001133
**P81265**	Polymeric immunoglobulin receptor	82.383	7,733333333	1	7,858686447
**A2I7N2**	Serpin A3-6	46.361	7,733333333	1	7,858686447
**K4JB97**	Alpha-2-macroglobulin variant 4	42.306	7,733333333	1	7,858686447
**A0A140T8A9**	Kappa-casein	21.224	1,431127013	1	4,702090144
**Q28107**	Coagulation factor V	248.828	15,10791367	1	11,06255352
**Q0P5J4**	Keratin. type I cytoskeletal 25	49.282	10	1	12,63318157
**A5D7J0**	ACTA2 protein	42.01	10	1	12,63318157
**P00366**	Glutamate dehydrogenas	61.473	10	1	12,63318157
**Q3ZC00**	Lymphocyte cytosolic protein 1 (L-plastin)	70.01	10	1	12,63318157
**Q148H6**	Keratin. type I cytoskeletal 28	50.744	27,06222865	1	453,5852154
**G8JKW7**	Uncharacterized protein	46.315	32,60869565	1	5,436547518
**Q9MZ31**	Fibronectin variable region (Fragment)	20.365	32,78688525	1	5,436547518
**Q2HJB8**	Tubulin alpha-8 chain	50.022	19,53125	1	5,436547518
**F1MYX5**	Lymphocyte cytosolic protein 1	70.068	32,78688525	1	5,436547518
**A6QNW3**	PIGR protein	82.455	9,693877551	1	5,790735483
**Q5XQN5**	Keratin. type II cytoskeletal 5	62.898	6,181015453	1	6,662236333
**P79334**	Glycogen phosphorylase	97.232	19,35483871	1	12,07949877
**Q3MHN2**	Complement component C9	61.958	16,02564103	1	5,436547518

*Classification given by author.
