# Supplementary material for: Extracellular vesicles in infectious diseases caused by protozoan parasites in buffaloes
Source: J Venom Anim Toxins Incl Trop Dis. 2020 May 29;26:e20190067. doi: 10.1590/1678-9199-JVATITD-2019-0067 (PMC7262785; doi:10.1590/1678-9199-JVATITD-2019-0067)
Supplement: Additional file 4. [file 1678-9199-jvatitd-26-e20190067-s4.pdf]

J Venom Anim Toxins incl Trop Dis
https://doi.org/10.1590/1678-9199-JVATITD-2019-0067

Supplementary Material to "Extracellular vesicles in infectious diseases caused by protozoan parasites in buffaloes"

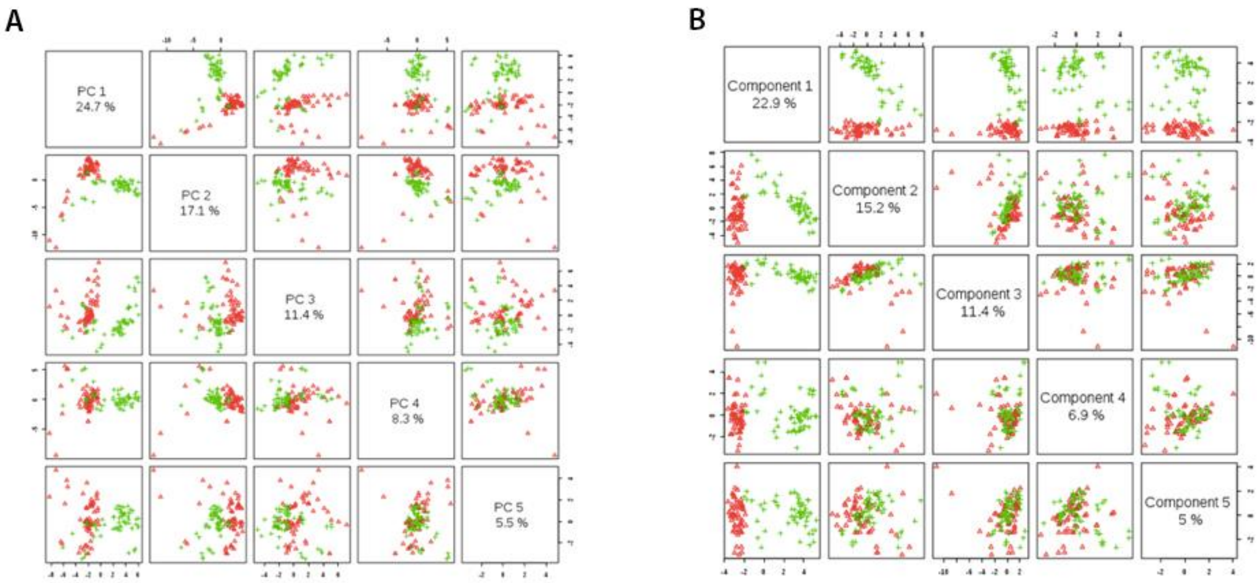

Additional file 4. Analysis of proteomic data in which control animals and *Theileria* spp. positive animals presented relevant statistical differences. **(A)** Principal component analysis (PCA). **(B)** Partial least squares (PSL).
