# Supplementary material for: Extracellular vesicles in infectious diseases caused by protozoan parasites in buffaloes
Source: J Venom Anim Toxins Incl Trop Dis. 2020 May 29;26:e20190067. doi: 10.1590/1678-9199-JVATITD-2019-0067 (PMC7262785; doi:10.1590/1678-9199-JVATITD-2019-0067)
Supplement: Additional file 5. [file 1678-9199-jvatitd-26-e20190067-s5.pdf]

J Venom Anim Toxins incl Trop Dis
https://doi.org/10.1590/1678-9199-JVATITD-2019-0067

Supplementary Material to "Extracellular vesicles in infectious diseases caused by protozoan parasites in buffaloes"

Additional file 5. Proteins identified with differential expression by fold change analysis

	Protein	Access code	Fold change	Log2 (FC)
1	Creatine (phospho) kinase	Q9TTK8	0.049467	-4.3419
2	L-lactate dehydrogenase	B0JYN3	0.091992	-3.5690
3	L-lactate dehydrogenase	Q5E9B1	0.12563	-3.2525
4	L-lactate	P19858	0.12367	-3.086
5	Homocysteine	Q5I597	0.13065	-2.6352
