# Supplementary material for: Extracellular vesicles in infectious diseases caused by protozoan parasites in buffaloes
Source: J Venom Anim Toxins Incl Trop Dis. 2020 May 29;26:e20190067. doi: 10.1590/1678-9199-JVATITD-2019-0067 (PMC7262785; doi:10.1590/1678-9199-JVATITD-2019-0067)
Supplement: Additional file 6. [file 1678-9199-jvatitd-26-e20190067-s6.pdf]

J Venom Anim Toxins incl Trop Dis
https://doi.org/10.1590/1678-9199-JVATITD-2019-0067

Supplementary Material to "Extracellular vesicles in infectious diseases caused by protozoan parasites in buffaloes"

Additional file 6. Proteins identified with differential expression by t-tests

	Protein	Access code	t.stat	p value	-log10 (p)	FDR
1	Creatine (phospho) kinase	Q9TTK8	-12.567	4.2136e-12	24.592	1.2465e-13
2	L-lactate dehydrogenase	B0JYN3	-12.782	2.1985e-15	20.986	2.1591e-10
3	L-lactate dehydrogenase	Q5E9B1	-11.696	2.1258e-11	21.512	2.1568e-11
4	L-lactate	P19858	-12.186	2.7674e-10	18.625	2.1025e-29
5	Homocysteine	Q5I597	-11.955	2.1563e-29	19.121	1.2157e-28
